# Disulfidptosis: A New Target for Parkinson’s Disease and Cancer

**DOI:** 10.3390/cimb46090600

**Published:** 2024-09-12

**Authors:** Tingting Liu, Xiangrui Kong, Jianshe Wei

**Affiliations:** Institute for Brain Sciences Research, School of Life Sciences, Henan University, Kaifeng 475004, China; ltt0808@henu.edu.cn (T.L.); 20060002@vip.henu.edu.cn (X.K.)

**Keywords:** Parkinson’s disease, disulfidptosis, pan-cancer, prognosis, immune infiltration

## Abstract

Recent studies have uncovered intriguing connections between Parkinson’s disease (PD) and cancer, two seemingly distinct disease categories. Disulfidptosis has garnered attention as a novel form of regulated cell death that is implicated in various pathological conditions, including neurodegenerative disorders and cancer. Disulfidptosis involves the dysregulation of intracellular redox homeostasis, leading to the accumulation of disulfide bonds and subsequent cell demise. This has sparked our interest in exploring common molecular mechanisms and genetic factors that may be involved in the relationship between neurodegenerative diseases and tumorigenesis. The Gene4PD database was used to retrieve PD differentially expressed genes (DEGs), the biological functions of differential expression disulfidptosis-related genes (DEDRGs) were analyzed, the ROCs of DEDRGs were analyzed using the GEO database, and the expression of DEDRGs was verified by an MPTP-induced PD mouse model in vivo. Then, the DEDRGs in more than 9000 samples of more than 30 cancers were comprehensively and systematically characterized by using multi-omics analysis data. In PD, we obtained a total of four DEDRGs, including *ACTB*, *ACTN4*, *INF2*, and *MYL6*. The enriched biological functions include the regulation of the NF-κB signaling pathway, mitochondrial function, apoptosis, and tumor necrosis factor, and these genes are rich in different brain regions. In the MPTP-induced PD mouse model, the expression of ACTB was decreased, while the expression of ACTN4, INF2, and MYL6 was increased. In pan-cancer, the high expression of ACTB, ACTN4, and MYL6 in GBMLGG, LGG, MESO, and LAML had a poor prognosis, and the high expression of INF2 in LIHC, LUAD, UVM, HNSC, GBM, LAML, and KIPAN had a poor prognosis. Our study showed that these genes were more highly infiltrated in Macrophages, NK cells, Neutrophils, Eosinophils, CD8 T cells, T cells, T helper cells, B cells, dendritic cells, and mast cells in pan-cancer patients. Most substitution mutations were G-to-A transitions and C-to-T transitions. We also found that miR-4298, miR-296-3p, miR-150-3p, miR-493-5p, and miR-6742-5p play important roles in cancer and PD. Cyclophosphamide and ethinyl estradiol may be potential drugs affected by DEDRGs for future research. This study found that *ACTB*, *ACTN4*, *INF2*, and *MYL6* are closely related to PD and pan-cancer and can be used as candidate genes for the diagnosis, prognosis, and therapeutic biomarkers of neurodegenerative diseases and cancers.

## 1. Introduction

Cancer is one of the most common causes of death; meanwhile, the incidence and prevalence of central nervous system diseases are also very high. There exist complex biological connections between cancer and neurodegenerative diseases. Aging, marked by the decline of essential physiological functions vital for survival and reproduction, is recognized as a significant risk factor for various diseases [1]. Cancer is associated with numerous characteristics, including sustained proliferative signaling, evasion of growth suppressors, resistance to cell death, attainment of replicative immortality, induction of angiogenesis, and activation of invasion and metastasis [2]. Notably, current research indicates that factors such as cellular energy dysregulation and evasion of immune destruction are correlated indicators. These characteristics are influenced by genomic instability, mutations, and/or tumor-promoting inflammation. The traits of neurodegenerative diseases encompass neuronal dysfunction and degeneration [3], impaired synaptic plasticity, and proteinopathies involving misfolded amyloid-β (Aβ) and tau proteins in Alzheimer’s disease (AD), as well as α-synuclein aggregates in Parkinson’s disease (PD) [4,5]. Additionally, they include progressive muscle atrophy or muscular dystrophy, which leads to memory deficits, cognitive impairments, and motor disorders [6]. PD, the second most prevalent neurodegenerative disorder after AD, affects 1–2% of the general population and is characterized by the progressive loss of dopaminergic neurons in the substantia nigra (SN) [7].

Epidemiological studies have demonstrated a significant antagonistic relationship between AD/PD and cancer. The likelihood of developing cancer after a diagnosis of AD/PD is decreased, and the opposite holds true as well [8]. Cellular apoptosis, or programmed cell death, is thought to play a critical role in the selective degeneration of dopaminergic neurons in the SN in PD. Accelerated neuronal death and excessive proliferation of cancer cells can be seen as two extremes of a common pathway in cell cycle regulation, leading to the hypothesis that the cancer risk in PD patients may be lower than in the general population [9]. The relationship between cancer and PD has been studied in over 20 clinical and epidemiological investigations. While some studies suggest a potential link between PD and an increased cancer risk [10,11], the majority of reports indicate a lower-than-expected incidence of cancer among PD patients [12]. One possible explanation is that PD and certain cancers may share common biological and pathological foundations or regulatory mechanisms, such as cell apoptosis, oxidative stress, inflammatory reactions, etc. Furthermore, some studies have suggested that certain cancer treatment drugs may have an impact on PD, potentially increasing the risk of PD in patients or worsening their symptoms.

Scholars in the life sciences have long been captivated by the investigation of programmed cell death mechanisms, including cuproptosis and ferroptosis, both of which are associated with “ion transport” [13]. Disulfidptosis is a newly identified form of cell death that diverges from traditional mechanisms such as apoptosis, necrosis, autophagy, NETosis, and pyroptosis [14]. This rapid cell death is triggered by the excessive accumulation of cysteine within the cell, leading to disulfide stress. The abnormal buildup of disulfides results in the formation of dysfunctional disulfide bonds among actin cytoskeleton proteins, causing the collapse of the actin network and ultimately cell death. Disulfidptosis was discovered by Professor Ganapathy-Kanniappan’s team at MD Anderson Cancer Center in the United States and Professor Chen Junjie’s research group in China [15]. Their study revealed that cells with high expression of Solute Carrier Family 7 Member 11 (SLC7A11) experience a previously uncharacterized form of cell death known as disulfidptosis under glucose deprivation, due to the abnormal accumulation of disulfide molecules [14]. The excessive buildup of disulfide molecules induces disulfide stress in actin cytoskeleton proteins, resulting in increased disulfide bond levels within actin filaments. This leads to filament contraction and the eventual disruption of the cellular skeletal structure, culminating in cell death. Inhibitors targeting specific cell death pathways have been employed in the treatment of various diseases, including neurodegenerative disorders [16]. The identification of the novel disulfidptosis mechanism, where cell death is initiated by disulfide bonds within the cellular skeleton, offers new potential targets for therapeutic intervention [17]. The abnormal accumulation of disulfides within cells creates disulfide stress, which may lead to elevated cellular toxicity [18,19]. Recently, a regulated cell death modality triggered by disulfides has been reported, termed “disulfidptosis” [15]. Unlike other programmed cell death pathways, disulfidptosis is mediated by the actin cytoskeleton’s sensitivity to disulfide stress, presenting a promising strategy for the treatment of various diseases [15].

Increasing evidence suggests an intriguing relationship between cancer and neurodegenerative diseases. A better understanding of the relationship between the two will provide new avenues for studying these age-related diseases. In this study, we first investigated the function and expression of disulfidptosis-related genes (DRGs) in PD, and we performed a prognostic analysis of DRGs in PD using PD data samples obtained from the GEO database (https://www.ncbi.nlm.nih.gov/geo/, accessed on 1 March 2024 ). Subsequently, we obtained all cancer sample data from the UCSC database (https://xenabrowser.net/, accessed on 1 March 2024) and analyzed the differential expression, prognosis, survival curves, immune infiltration, and single-cell types of DRGs in cancer, aiming to elucidate the roles of DRGs in PD and tumors and to assist personalized treatment for PD and cancer.

## 2. Materials and Methods

### 2.1. Data Acquisition

PD-related differential expression genes (DEGs) were obtained from the gene4PD database (http://www.genemed.tech/gene4pd/, accessed on 5 March 2024). In addition, literature has been collected on genes associated with DRGs [15]. The “venneuler” package in R 4.2.0 software, ggplot2 [3.3.6], VennDiagram [1.7.3], was adopted to draw the intersection of DEGs and DRGs, i.e., differential expression disulfidptosis-related genes (DEDRGs). The KEGG pathway and GO enrichment of DEDRGs were analyzed utilizing the gene4PD database, and we used Cytoscape 3.10.1 to draw a network diagram. And we analyzed the average expression levels of DEDRGs utilizing the gene4PD database in different brain regions at different developmental periods using “BrainSpan” under “Gene expression” and the average expression level of DEDRGs in different tissue using “GTEx” under “Gene expression”.

### 2.2. Identifying the Diagnostic Value of DEDRGs

The receiver operating characteristic (ROC) curve is a graphical representation of the diagnostic value of a gene in predicting a certain condition or disease. It plots the true positive rate (sensitivity) against the false positive rate (1-specificity) at different threshold values. To screen the diagnostic genes, we visually displayed the ROC curve analysis, and the AUCs were calculated using the pROC package in R 4.2.1 software, pROC [1.18.0], ggplot2 [3.3.6], to determine the predicted values of the hub genes. We selected PD datasets from the GEO database, including GSE49036, GSE20163, GSE20164, GSE7621, GSE99039, and GSE22491. Diagnostic genes were selected from the set using the criterion of AUC > 0.500.

### 2.3. In Vivo Experiment Verification

#### 2.3.1. Animal

Experiments were conducted using male C57BL/6J mice aged 6–8 weeks, weighing between 20 and 25 g, provided by Cyagen Biosciences. The animals were maintained under standard conditions (12 h light/dark cycle, 22 ± 2 °C, and relative humidity of 55 ± 5%) with ad libitum access to food and water. All animal procedures adhered to the Guide for the Care and Use of Laboratory Animals and received approval from the Institutional Animal Care and Use Committee at Henan University. The experimenters were blinded to the mouse assignments. Prior to the experiment, the animals were housed in our facilities for two weeks to acclimate, after which they were randomly divided into two groups (3 mice in each group): a saline control group and a model group (1-methyl-4-phenyl-1,2,3,6-te-trahydropyridine (MPTP) (abmole, Houston, TX, USA, M9049), 20 mg/kg/d, administered via intraperitoneal injection for 5 days).

#### 2.3.2. Western Blot

SN was suspended in RIPA lysis solution containing PMSF (catalog # G2002 and # G2008, Servicebio, Wuhan, China) and then subjected to sonication and centrifuged at 12,000 rpm for 10 min at 4 °C, followed by the collection of the supernatant. The protein concentration in the supernatant was determined using the BCA assay, and samples were separated on 8%, 10%, and 12% SDS-PAGE gels before being transferred onto a nitrocellulose membrane (Millipore, IPFL00010, Darmstadt, Germany) via electrophoresis. The membrane was blocked with 5% nonfat milk in TBST for 1 h at room temperature and probed overnight at 4 °C with primary antibodies: beta Actin (1:3000, AF7018, Affinity, Melbourne, Australia), ACTN4 (1:1000, DF8000, Affinity, Melbourne, Australia), INF2 (1:1500, 20466-1-AP, Proteintech, Chicago, IL, USA), MYL6 (1:1000, DF4718, Affinity, Melbourne, Australia), and GAPDH (1:3000, Affinity, Melbourne, Australia) in TBST containing 1% nonfat milk. Following the overnight incubation, the membrane was incubated for 2 h at room temperature with an HRP-conjugated secondary antibody in TBST with 1% nonfat milk. The blots were developed using an Enhanced Chemiluminescence assay (BIO-Rad, 1708280, Hercules, CA, USA). Densitometric analysis of the scanned Western blot images was performed using ImageJ software Version 1.54j.

#### 2.3.3. DEDRG Differential Expression of Pan-Cancer

We used the Sanger Box (http://sangerbox.com/, accessed on 8 March 2024) website to analyze the differential expression of DEDRGs in pan-cancer, as follows: We downloaded the uniformly normalized pan-cancer dataset, TCGA Pan-Cancer (PANCAN, N = 10,535, G = 60,499), from the UCSC database. We further screened samples from Solid Tissue Normal, Primary Blood Derived Cancer Peripheral Blood, and Primary Tumor. We performed a log2 (x + 0.001) transformation on each expression value. Additionally, we excluded cancer types with fewer than 3 samples in a single cancer type, resulting in expression data for 26 cancer types. Next, we extracted the expression data of the genes ENSG0000075624 (ACTB), ENSG000000130402 (ACTN4), ENSG0000092841 (MYL6), and ENSG0000003485 (INF2) from each sample. Using R software (version 3.6.4), we calculated the expression differences between normal and tumor samples in each tumor, using non-paired Wilcoxon Rank Sum and Signed Rank Tests for differential significance analysis.

#### 2.3.4. DEDRG Prognostic Analysis of Pan-Cancer

We used the Sanger Box (http://sangerbox.com/, accessed on 9 March 2024) website for the prognostic analysis of DEDRGs in pan-cancer, as follows: We downloaded the uniformly normalized pan-cancer dataset from the UCSC database. Additionally, we filtered samples from the following sources: Primary Blood Derived Cancer Peripheral Blood (TCGA-LAML), Primary Tumor, and Metastatic samples from TCGA-SKCM. We also acquired high-quality prognostic data for TCGA from a previously published study in *Cell* [20]. Samples with follow-up times of less than 30 days were excluded. Each expression value was transformed using a log2 (x + 0.001) transformation. Moreover, we removed cancer types with fewer than 10 samples, leading to expression data and corresponding overall survival data for 39 cancer types. We utilized the ‘coxph’ function from the R package ‘survival’ (version 3.2-7) to establish Cox proportional hazards regression models and analyze the relationship between gene expression and prognosis in each tumor. We conducted statistical tests using the Logrank test to determine prognostic significance.

#### 2.3.5. Survival Curve Analysis of Pan-Cancer

RNA sequencing (RNAseq) data were obtained from The Cancer Genome Atlas (TCGA) database, accessible via the GDC portal (https://portal.gdc.cancer.gov, accessed on 12 March 2024). We focused on cancer projects that exhibited significant differences in prognosis. To ensure robust analysis, we extracted the data in Transcripts Per Million (TPM) format alongside relevant clinical information sourced from a *Cell* article [20]. Normal samples and those with incomplete clinical data were systematically excluded to maintain the integrity of our analysis. To investigate the relationship between gene expression and patient survival, we employed Cox proportional hazards regression analysis using the ‘survival’ package in R (version 4.2.1). Prior to analysis, we conducted tests for the proportional hazards assumption to validate the suitability of the data for this statistical method. Survival curves derived from the Cox models were visualized using the ‘survminer’ and ‘ggplot2’ packages (version 3.3.6) to illustrate the survival outcomes across different cancer types. This approach facilitated an intuitive representation of survival differences based on the prognostic analysis. To further corroborate our findings regarding the variability in survival curves across cancer types, we utilized the GEPIA database (http://gepia.cancer-pku.cn/, accessed on 17 March 2024) for additional verification. This validation step was critical to substantiate our results and ensure the robustness of the observed survival disparities.

#### 2.3.6. Immune Infiltration and Single-Cell Type Analysis

Based on the ssGSEA algorithm provided in the R package GSVA [1.46.0] [21], we utilized the markers of 24 immune cells provided in an *Immunity* article to calculate the immune infiltration status of the corresponding cloud-based data [22]. We conducted a correlation analysis between the main variables in the data and the immune infiltration matrix data, and we visualized the analysis results using the ggplot2 [3.3.6] package with a lollipop chart. Furthermore, we performed cellular localization analysis of DEDRGs, analyzed their expression in different tissues, and investigated their pathological manifestations in different cancers using the Human Protein Atlas (HPA) database (https://www.proteinatlas.org/, accessed on 19 March 2024).

#### 2.3.7. Mutation and miRNA Analysis of DEDRGs

We utilized the cBioPortal database (https://www.cbioportal.org/, accessed on 21 March 2024) to analyze the mutation status and mutation sites of DEDRGs in different types of cancer. We used the COSMIC database (https://cancer.sanger.ac.uk/cosmic, accessed on 22 March 2024) to analyze the mutation types of DEDRGs in somatic cells. We predicted miRNAs associated with DEDRGs using the miRWalk database (http://mirwalk.umm.uni-heidelberg.de/, accessed on 26 March 2024), and we created a “gene–miRNA” network diagram using Cytoscape 3.10.1.

#### 2.3.8. Molecular Docking and Prediction of Binding Pockets

A gene–drug interaction network was constructed to identify potential new targets using the DGldb database (https://dgidb.org/, accessed on 27 March 2024). To analyze the binding affinities and interaction modes between the drug candidates and their targets, Autodock Vina 1.2.2, a computational protein–ligand docking software, was employed [23]. Molecular structures were obtained from the PubChem database (https://pubchem.ncbi.nlm.nih.gov, accessed on 28 March 2024). The PDB database (https://www.rcsb.org/, accessed on 29 March 2024) was used for retrieving protein structures in PDB format. For docking analysis, all protein and molecular files were converted to PDBQT format, with water molecules excluded and polar hydrogen atoms added. The grid box was centered to encompass the domain of each protein, allowing free movement of the molecules. The dimensions of the grid box were set at 30 Å × 30 Å × 30 Å, with a grid point distance of 0.05 nm. Molecular docking studies were carried out using Autodock Vina 1.2.2 (http://autodock.scripps.edu/, accessed on 30 March 2024). The DoGSiteScorer database (https://proteins.plus/, accessed on 30 March 2024) was used to predict protein-binding sites.

#### 2.3.9. Statistical Analysis

Data are presented as the mean ± SEM, and comparisons between groups were performed using unpaired Wilcoxon Rank Sum and Signed Rank Tests. Significant differences were statistically analyzed using the Logrank test. ROCs were used to evaluate AUCs and predictive abilities. A *p*-value of less than 0.05 was considered statistically significant.

## 3. Results

### 3.1. Biological Functional Enrichment Analysis of DEDRGs

**Figure 1:**
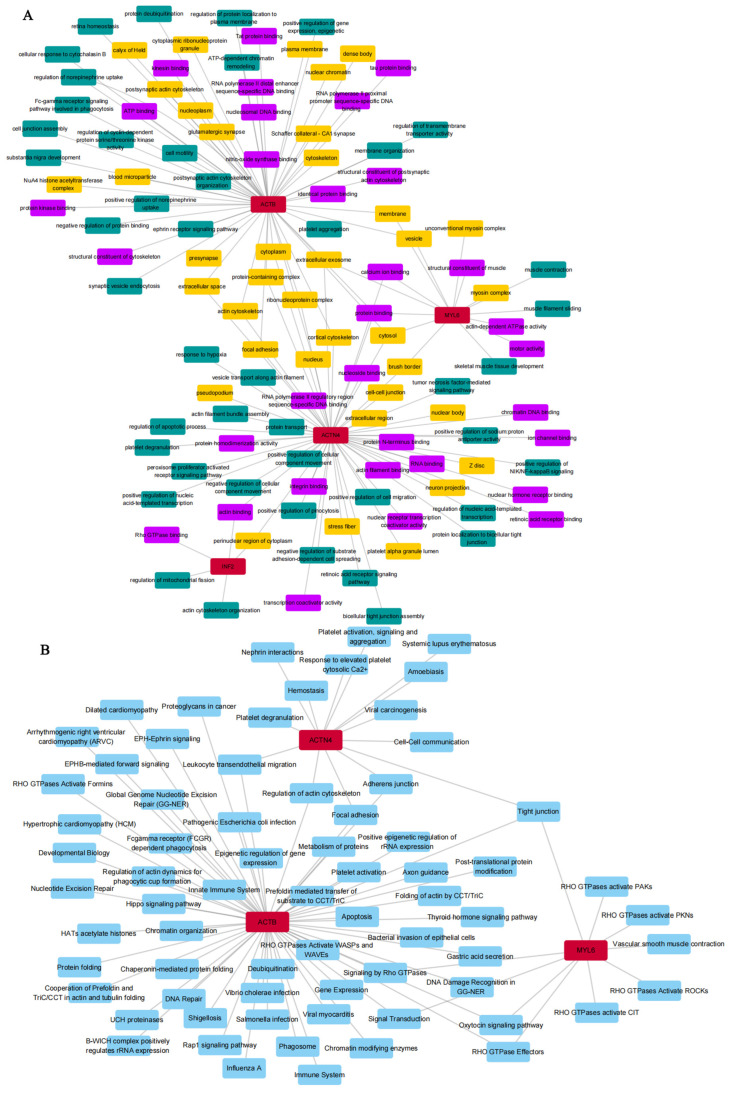
The DEDRG-enriched GO terms and KEGG pathways. (**A**) GOBP, GOCC, and GOMF analysis. (**B**) Signaling pathway enrichment analysis. Red represents DEDRGs, green represents biological process, purple represents molecular function, orange represents cellular component, and blue represents signaling pathways.

Ultimately, combined with DEGs and DRGs, we screened four overlapping genes (DEDRGs) for further study (Supplementary Figure S1). Gene Ontology and KEGG analyses were conducted to reveal the possible biological functions and enrichment pathways of DEDRGs. The GO analysis was categorized into biological processes (BPs), cell components (CCs), and molecular functions (MFs) (Figure 1A). The BP of DEDRGs was mainly enriched in positive regulation of NIK/NF-kappaB signaling, negative regulation of substrate adhesion-dependent cell spreading, bicellular tight junction assembly, positive regulation of cellular component movement, regulation of mitochondrial fission, regulation of the apoptotic process, the peroxisome proliferator-activated receptor signaling pathway, tumor necrosis factor-mediated signaling pathway, the ephrin receptor signaling pathway, positive regulation of gene expression, epigenetic, and ATP-dependent chromatin remodeling. The CC of DEDRGs was mainly enriched in actin cytoskeleton, focal adhesion, plasma membrane, cytoskeleton, cytosol, cytoplasm, nucleoplasm, ribonucleoprotein complex, glutamatergic synapse, postsynaptic actin cytoskeleton, blood microparticle, extracellular exosome, perinuclear region of cytoplasm, and neuron projection. The MF of DEDRGs was mainly enriched in nitric-oxide synthase binding, tau protein binding, RNA binding, transcription coactivator activity, ion channel binding, retinoic acid receptor binding, nuclear receptor transcription coactivator activity, calcium ion binding, Rho GTPase binding, and actin-dependent ATPase activity. KEGG mainly includes DNA repair, regulation of cell apoptosis, regulation of the immune system, signal transduction of Rho GTPases, protein folding, metabolism, modification, platelet activation, the Hippo signaling pathway, the Rap1 signaling pathway, platelet activation, signaling and aggregation, leukocyte transient migration, vascular smooth muscle competition, etc. (Figure 1B).

### 3.2. Analysis of DEDRG Expression in Tissues

**Figure 2:**
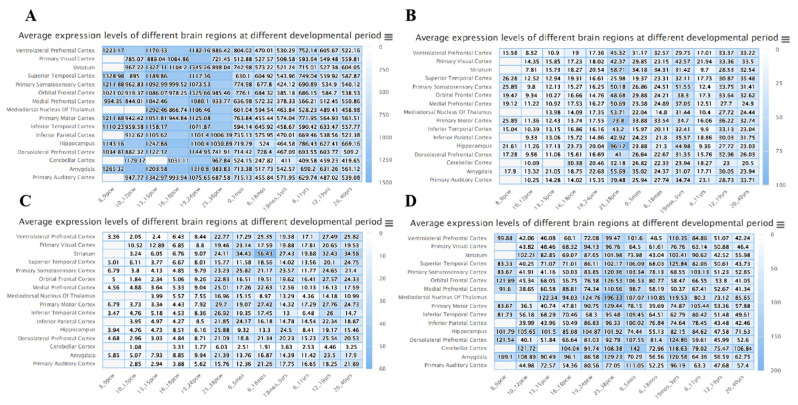
Spatio-temporal expression profiles of (**A**) ACTB, (**B**) ACTN4, (**C**) INF2, and (**D**) MYL6 retrieved from BrainSpan. The darker the blue color, the higher the protein expression level in the brain region.

ACTB is highly expressed in all tissues except bone marrow. ACTN4 is expressed at low levels in muscle, brain, testis, heart, pituitary, liver, blood, pancreas, and bone marrow. INF2 is expressed at low levels in all tissues except nerve. MYL6 is expressed at low levels in muscle and bone marrow (less than 50 RPKM) (Supplementary Figure S2). Furthermore, we observed that proteins are expressed differently in various tissues during the fetal stage and adulthood. For example, ACTB expression is highest in cord blood during the fetal period, while it is highest in blood during adulthood (Supplementary Figure S3). In the brain, ACTB is highly expressed in different brain regions and stages (Figure 2A). ACTN4 shows higher protein levels in the hippocampus, with elevated levels in different brain regions during post-conception weeks (PCWs) 28–35 and 19 months to 5 years, stabilizing from youth to adulthood (Figure 2B). INF2 exhibits higher levels in the striatum, with levels increasing and stabilizing after 28–35 PCWs (Figure 2C). MYL6 is present in different brain regions and highly expressed after 10–12 PCWs, with a slight decrease in the expression trend from 12 to 19 years (Figure 2D). ACTB, ACTN4, INF2, and MYL6 are involved in important biological processes such as cell morphology, synaptic regulation, cell movement, and synaptic plasticity in the brain [24,25,26,27].

### 3.3. Identifying the Diagnostic Value of DEDRGs

**Figure 3:**
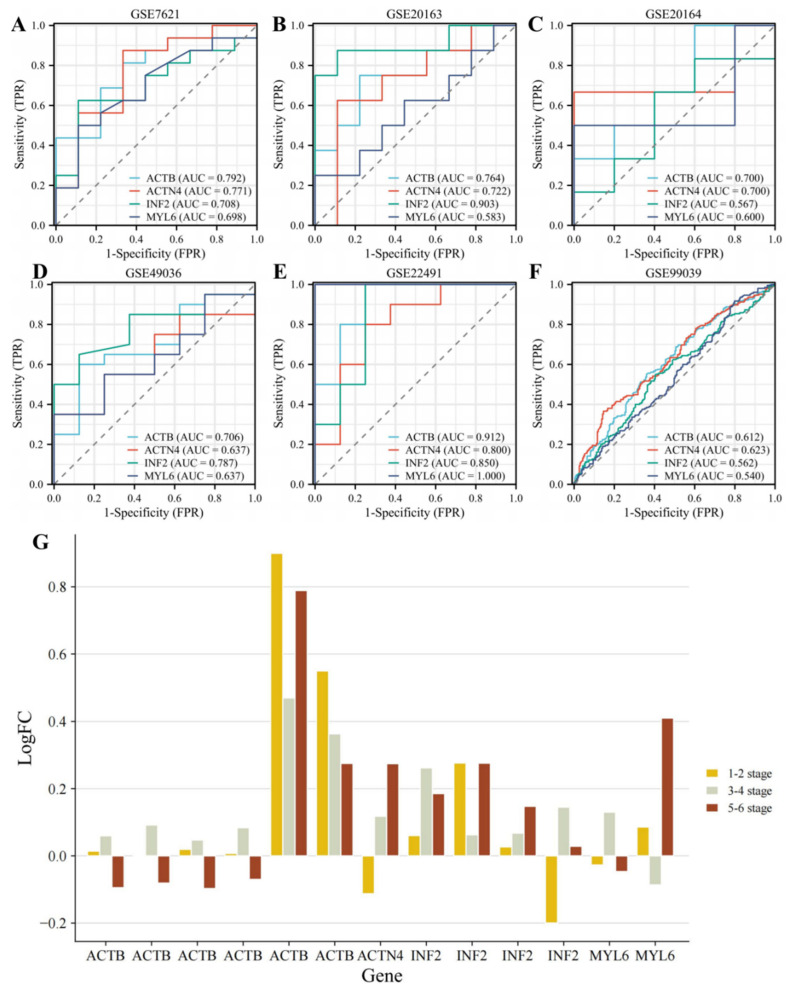
Diagnostic value of DEDRGs in (**A**) 16 PD and 9 control subjects from the substantia nigra postmortem brain from the GSE7621 dataset; (**B**) 8 PD and 9 control subjects from the substantia nigra of postmortem brains from the GSE20163 dataset; (**C**) 6 PD and 5 control subjects from substantia nigra samples from the GSE20164 dataset; (**D**) control Braak α-synuclein Stage 0: 8 samples; Braak α-synuclein stages 1–2: 5 samples; Braak α-synuclein stages 3–4: 7 samples; Braak α-synuclein stages 5–6: 8 samples from the GSE49036 dataset; (**E**) 8 PD and 8 control subjects from peripheral mononuclear blood cells from the GSE22491 dataset; (**F**) 233 healthy controls and 205 idiopathic PD patients from whole blood from the GSE99039 dataset. (**G**) The expression of DEDRGs from the GSE49036 dataset at different stages. Gene ID: 200801_x_at, 213867_x_at, 224594_x_at, AFFX-HSAC07/X00351_3_at, AFFX-HSAC07/X00351_5_at, AFFX-HSAC07/X00351_M_at, 200601_at, 218144_s_at, 222534_s_at, 222535_at, 224469_s_at, 212082_s_at, 214002_at.

By analyzing the ROC curve, we can determine the performance of a gene as a diagnostic marker. The closer the curve is to the top left corner of the plot, the higher the diagnostic accuracy. A high AUC (>0.5) indicates that the gene has potential diagnostic value. In the PD dataset, we found that the AUC values were greater than 0.5 for both substantia nigra and peripheral blood samples (Figure 3A–F), indicating that DEDRGs have potential diagnostic value and could serve as potential biomarkers for treating PD. Subsequently, we analyzed the expression of DEDRGs in PD using the GSE49036 dataset. Based on Braak staging, PD was divided into stages 1–2, 3–4, and 5–6. We observed that as the aggregation of α-syn increased, the expression of ACTB decreased, while the expression of ACTN4, INF2, and MYL6 increased (Figure 3G).

**Figure 4:**
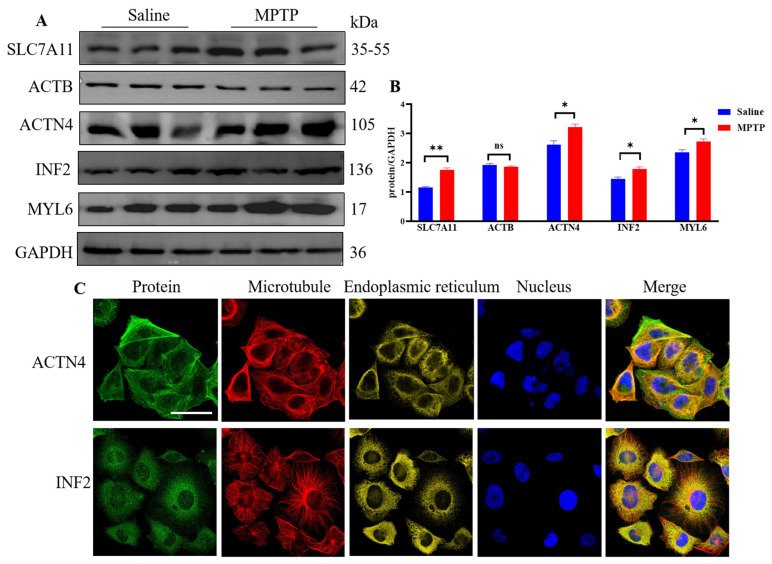
Validity verification of DEDRGs. (**A**) Validation of DEDRGs by Western blotting. (**B**) Statistical plots of SLC7A11, ACTB, ACTN4, INF2, and MYL6. Compared with the saline group, ns = no significance, * *p <* 0.05, ** *p* < 0.01. *n* = 3. (**C**) Location of ACTN4 and INF2 proteins in cells from the HPA database: green represents the target protein, red represents microtubules, yellow represents the endoplasmic reticulum, and blue represents the nucleus (scale bar, 20 µm).

Furthermore, experimental validation of the DEDRGs showed that compared with the saline group, the expression of ACTN4, INF2, and MYL6 increased in the MPTP group (*p* < 0.05), with ACTB showing a non-significant decrease (Figure 4A,B), although the differences were not statistically significant, likely due to the small sample size. High expression of SLC7A11 leads to significant depletion of intracellular NADPH and abnormal accumulation of cysteine and other disulfide compounds, inducing disulfide stress and rapid cell death. In this study, compared with the saline group, the expression of SLC7A11 increased in the MPTP group (*p* < 0.01). This sufficiently indicates that DEDRGs have the potential to serve as biomarkers for PD. Finally, an analysis of the distribution of ACTN4 and INF2 in cells using the HPA database revealed that ACTN4 is mainly localized to the actin filaments and additionally to the cytosol, while INF2 is primarily localized to the endoplasmic reticulum and additionally to the nuclear bodies (Figure 4C).

### 3.4. DEDRG Differential Expression Analysis of Pan-Cancer

**Figure 5:**
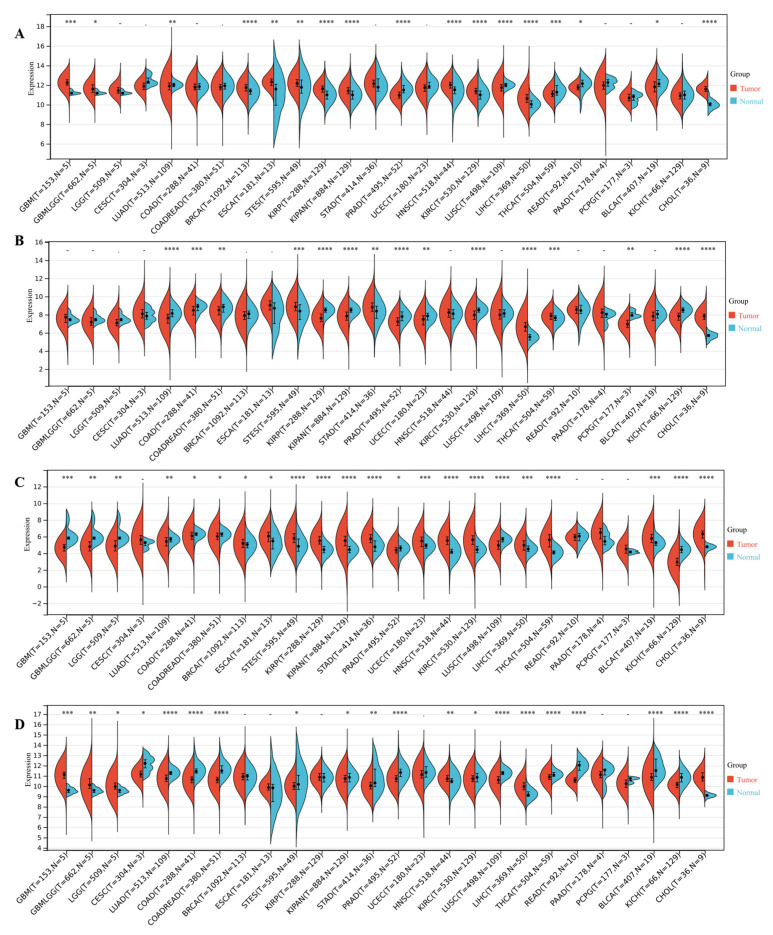
Box plot of differential expression of DEDRGs between normal and tumor samples. (**A**) The differential expression of ACTB in pan-cancer. (**B**) The differential expression of ACTN4 in pan-cancer. (**C**) The differential expression of INF2 in pan-cancer. (**D**) The differential expression of MYL6 in pan-cancer. Compared with the normal samples, * *p* < 0.05, ** *p* < 0.01, *** *p* < 0.001, **** *p* < 0.0001.

ACTB was significantly upregulated in 11 types of tumors, namely GBM, GBMLGG, BRCA, ESCA, STES, KIRP, KIPAN, HNSC, KIRC, LIHC, and CHOL, and significantly downregulated in 6 types of tumors, namely LUAD, PRAD, LUSC, THCA, READ, and BLCA (Figure 5A). ACTN4 was significantly upregulated in 5 types of tumors, namely STES, STAD, LIHC, THCA, and CHOL, and significantly downregulated in 10 types of tumors, namely LUAD, COAD, COADREAD, KIRP, KIPAN, PRAD, UCEC, KIRC, PCPG, and KICH (Figure 5B). INF2 was significantly upregulated in 13 types of tumors, namely BRCA, ESCA, STES, KIRP, KIPAN, STAD, UCEC, HNSC, KIRC, LIHC, THCA, BLCA, and CHOL, and significantly downregulated in 9 types of tumors, namely GBM, GBMLGG, LGG, LUAD, COAD, COADREAD, PRAD, LUSC, and KICH (Figure 5C). MYL6 was significantly upregulated in 6 types of tumors, namely GBM, GBMLGG, LGG, HNSC, LIHC, and CHOL, and significantly downregulated in 14 types of tumors, namely CESC, LUAD, COAD, COADREAD, STES, KIPAN, STAD, PRAD, KIRC, LUSC, THCA, READ, BLCA, and KICH (Figure 5D). The expression values of tumor samples and normal samples are presented in Supplementary Table S1. The abbreviation table for cancer is shown in Table 1.

### 3.5. DEDRG Prognostic Analysis of Pan-Cancer

**Figure 6:**
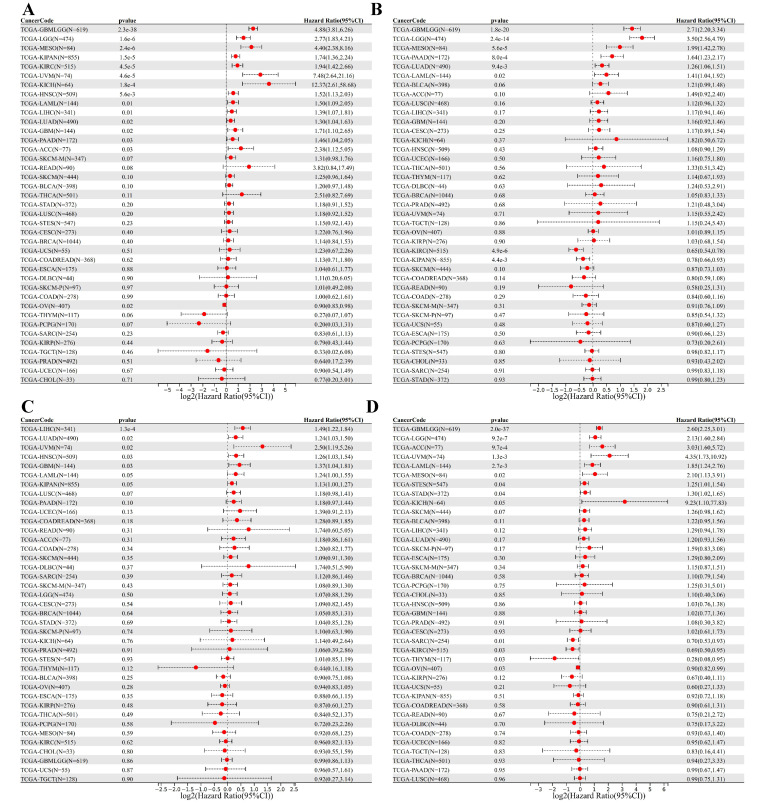
Pan-cancer prognostic analysis of DEDRGs using univariate Cox regression, including (**A**) ACTB, (**B**) ACTN4, (**C**) INF2, and (**D**) MYL6.

Prognostic analysis in tumors involves evaluating various factors to assess the likelihood of disease progression, response to treatment, and overall patient outcome. ACTB has a poor prognosis with high expression in 14 tumor types (GBMLGG, LGG, MESO, KIPAN, KIRC, UVM, KICH, LAML, HNSC, LIHC, LUAD, GBM, PAAD, ACC) and a poor prognosis with low expression in 1 tumor type (OV) (Figure 6A). ACTN4 has a poor prognosis with high expression in six tumor types (GBMLGG, LGG, MESO, PAAD, LUAD, LAML) and a poor prognosis with low expression in two tumor types (KIPAN, KIRC) (Figure 6B). INF2 has a poor prognosis with high expression in seven tumor types (LIHC, LUAD, UVM, HNSC, GBM, LAML, KIPAN) (Figure 6C). MYL6 has a poor prognosis with high expression in eight tumor types (GBMLGG, LGG, ACC, UVM, LAML, MESO, STES, STAD) and a poor prognosis with low expression in four tumor types (SARC, KIRC, THYM, OV) (Figure 6D). Tumor expression data and overall survival data of samples are shown in Supplementary Table S2.

### 3.6. Survival Curve Analysis of Pan-Cancer

**Figure 7:**
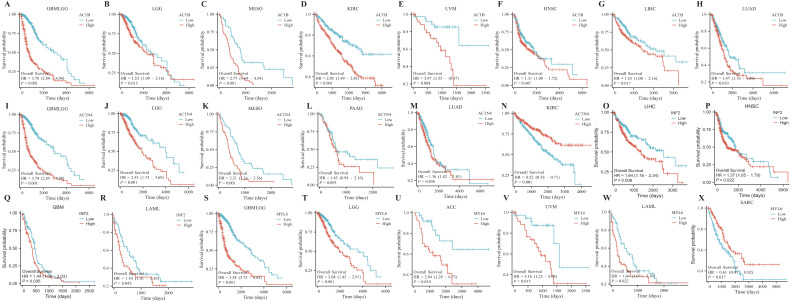
Survival analysis of DEDRG expression in pan-cancer. (**A**–**H**) Survival curves of ACTB in GBMLGG, LGG, MESO, KIRC, UVM, HNSC, LIHC, LUAD. (**I**–**N**) Survival curves of ACTN4 in GBMLGG, LGG, MESO, PAAD, LUAD, KIRC. (**O**–**R**) Survival curves of INF2 in LIHC, HNSC, GBM, LAML. (**S**–**X**) Survival curves of MYL6 in GBMLGG, LGG, ACC, UVM, LAML, SARC.

ACTB has a long survival period with low expression in GBMLGG, LGG, MESO, KIRC, UVM, HNSC, LIHC, and LUAD (Figure 7A–H). ACTN4 has a long survival period with low expression in GBMLGG, LGG, MESO, PAAD, and LUAD (Figure 7I–M) and a long survival period with high expression in KIRC (Figure 7N). INF2 has a long survival period with low expression in LIHC, HNSC, GBM, and LAML (Figure 7O–R). MYL6 has a long survival period with low expression in GBMLGG, LGG, ACC, UVM, and LAML (Figure 7S–W) and a long survival period with high expression in SARC (Figure 7X). We also used the GEPIA database to verify the impact of gene expression on the survival of tumor patients and obtained results consistent with the above (Supplementary Figure S4). The survival curve shows no significant difference in Supplementary Figure S5.

### 3.7. Immune Infiltration Analysis

**Figure 8:**
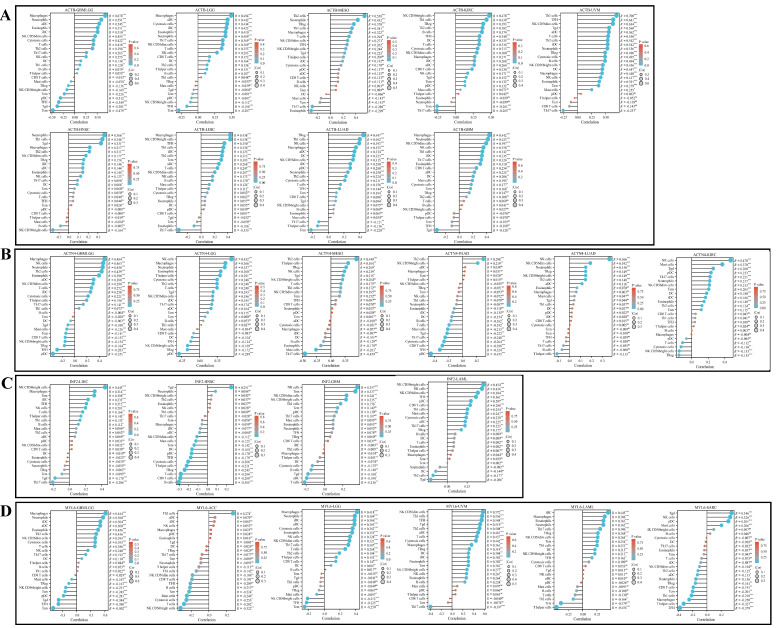
Pan-cancer immune infiltration analysis: (**A**) Immunoinfiltration analysis of ACTB in GBMLGG, LGG, MESO, KIRC, UVM, HNSC, LIHC, LUAD, and GBM. (**B**) Immunoinfiltration analysis of ACTN4 in GBMLGG, LGG, MESO, PAAD, LUAD, and KIRC. (**C**) Immunoinfiltration analysis of INF2 in LIHC, HNSC, GBM, and LAML. (**D**) Immunoinfiltration analysis of MYL6 in GBMLGG, ACC, LGG, UVM, LAML, and SARC. The correlation coefficient being positive indicates a positive correlation between two variables; a negative correlation coefficient indicates a negative correlation between two variables. The absolute value of the correlation coefficient represents the degree of correlation: 0–0.3 indicates weak or no correlation; 0.3–0.5 indicates weak correlation; 0.5–0.8 indicates moderate correlation; 0.8–1 indicates strong correlation. * *p* < 0.05, ** *p* < 0.01, *** *p* < 0.001.

Immune infiltration analysis can help us understand the type, quantity, distribution, and function of immune cells in tumor tissue, thereby leading to a deeper understanding of the interaction between tumors and the immune system. For example, ACTB is positively correlated with Macrophages, Neutrophils, aDC, Eosinophils, iDC, natural killer (NK) CD56dim cells, cytotoxic cells, T cells, Th2 cells, Th17 cells, NK cells, DC, Thl cells, and B cells and negatively correlated with mast cells, TReg, NK CD56bright cells, Tem, pDC, TFH, Tgd, and Tcm in GBMLGG (Figure 8A). ACTN4 is positively correlated with Macrophages, NK cells, Neutrophils, Th2 cells, Eosinophils, aDC, NK CD56dim cells, T cells, iDC, cytotoxic cells, T helper cells, and Th17 cells and negatively correlated with Tgd, mast cells, Tcm, CD8 T cells, NK CD56bright cells, TReg, TFH, and pDC in GBMLGG (Figure 8B). INF2 is positively correlated with NK CD56bright cells, Macrophages, Tem, iDC, TFH, NK cells, T cells, T helper cells, Th1 cells, and B cells and negatively correlated with Tgd and Th17 cells in LIHC (Figure 8C). MYL6 is positively correlated with Macrophages, Neutrophils, iDC, aDC, Eosinophils, Th2 cells, NK CD56dim cells, cytotoxic cells, T cells, NK cells, Th17 cells, and DC and negatively correlated with mast cells, TReg, NK CD56bright cells, Tem, pDC, Tgd, TFH, and Tcm (Figure 8D).

### 3.8. Single-Cell Type Analysis

**Figure 9:**
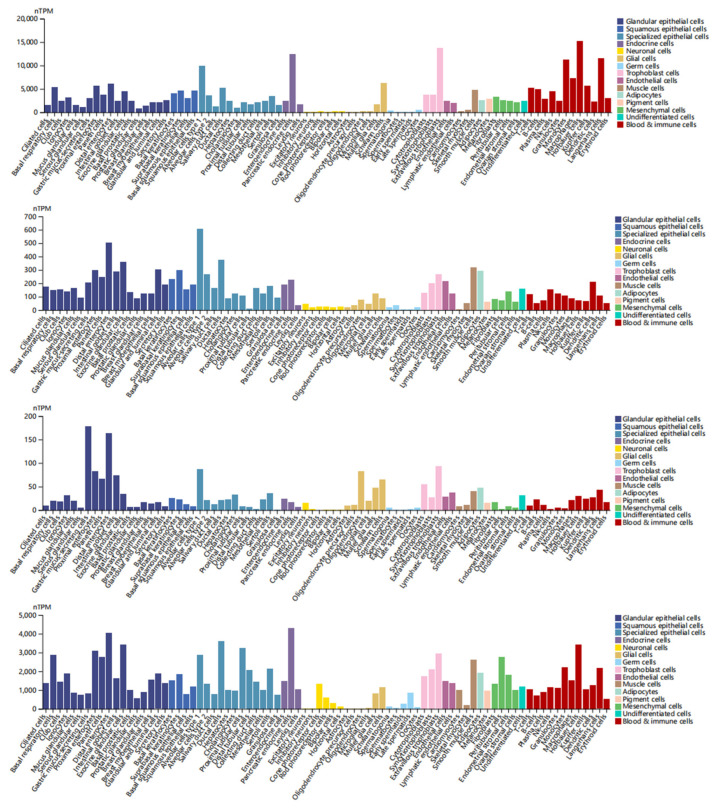
Single-cell type analysis of DEDRGs, including ACTB, ACTN4, INF2, and MYL6, mainly from glandular epithelial cells, squamous epithelial cells, specialized epithelial cells, endocrine cells, neuronal cells, glial cells, germ cells, trophoblast cells, endothelial cells, muscle cells, adipocytes, pigment cells, mesenchymal cells, undifferentiated cells, and blood and immune cells.

In single-cell type analysis, we analyzed the expression of DEDRGs mainly from glandular epithelial cells, squamous epithelial cells, specialized epithelial cells, endocrine cells, neuronal cells, glial cells, germ cells, trophoblast cells, endothelial cells, muscle cells, adipocytes, pigment cells, mesenchymal cells, undifferentiated cells, and blood and immune cells. ACTB is highly expressed in various cells, including distal enterocytes, suprabasal keratinocytes, alveolar cells type 1, pancreatic endocrine cells, horizontal cells, Schwann cells, oocytes, extravillous trophoblasts, endothelial cells, smooth muscle cells, adipocytes, melanocytes, fibroblasts, undifferentiated cells, and Hofbauer cells (Figure 9). ACTN4 is highly expressed in various cells, including distal enterocytes, suprabasal keratinocytes, type 1alveolar cells, pancreatic endocrine cells, excitatory neurons, Muller glia cells, spermatocytes, extravillous trophoblasts, endothelial cells, smooth muscle cells, adipocytes, melanocytes, endometrial stromal cells, undifferentiated cells, and dendritic cells (Figure 9). INF2 is highly expressed in various cells, including gastric mucus-secreting cells, type 1 alveolar cells, enteroendocrine cells, excitatory neurons, oligodendrocytes, spermatogonia, extravillous trophoblasts, lymphatic endothelial cells, smooth muscle cells, adipocytes, melanocytes, fibroblasts, undifferentiated cells, and Langerhans cells (Figure 9). MYL6 is highly expressed in various cells, including distal enterocytes, suprabasal keratinocytes, ductal cells, pancreatic endocrine cells, cone photoreceptor cells, Schwann cells, late spermatids, extravillous trophoblasts, endothelial cells, smooth muscle cells, adipocytes, melanocytes, peritubular cells, undifferentiated cells, and Hofbauer cells (Figure 9). A single-cell tissue overview is shown in Supplementary Figure S6.

### 3.9. Gene Mutation Analysis of DEDRGs

**Figure 10:**
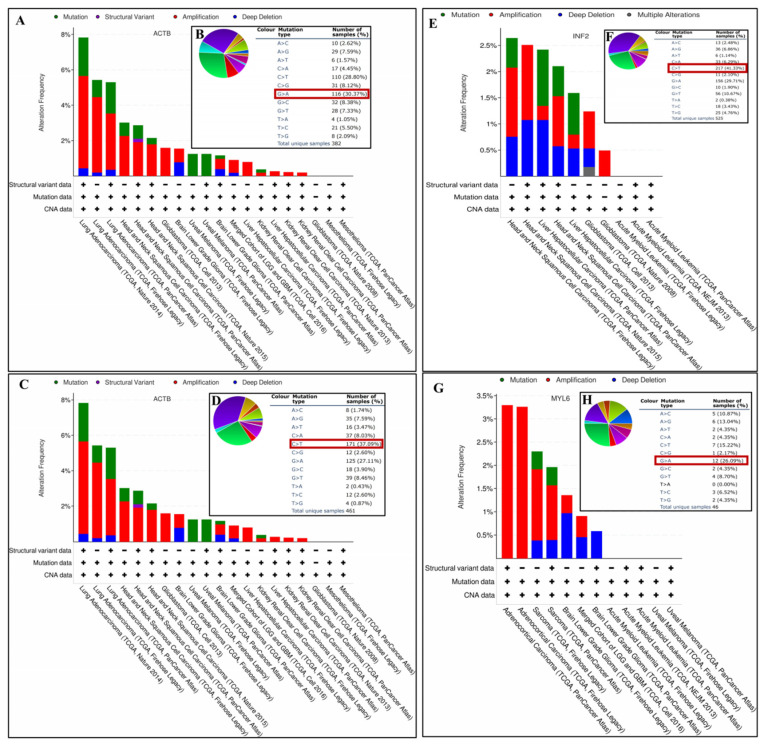
Gene mutation analysis of DEDRGs. (**A**) The pan-cancer mutation status of ACTB was determined using the cBioPortal tool. (**B**) ACTB base mutation frequency. (**C**) The pan-cancer mutation status of ACTN4 was determined using the cBioPortal tool. (**D**) ACTN4 base mutation frequency. (**E**) The pan-cancer mutation status of INF2 was determined using the cBioPortal tool. (**F**) INF2 base mutation frequency. (**G**) The pan-cancer mutation status of MYL6 was determined using the cBioPortal tool. (**H**) MYL6 base mutation frequency.

Genetic mutations may lead to the loss of tumor suppressive function and DNA repair defects, activating the pro-cancer signaling pathway. Therefore, we analyzed the mutation of genes in tumors. The main mutation of *ACTB* in GBMLGG is Amplification (0.73%), LGG is Deep Deletion and Amplification (0.78%), KIRC is Mutation and Amplification (0.19%), UVM is Mutation (1.25%), HNSC is Amplification (2.26%, 1.91%, 1.91%), LIHC is Amplification (0.8%, 0.27%), LUAD is Amplification (5.22%, 4.26%, 3.18%), and GBM is Amplification (1.59%, 0.2%) (Figure 10A). Most substitution mutations were G-to-A transitions (30.37%) (Figure 10B). As shown in Supplementary Figure S7A, there were 35 mutations in the full sequence of *ACTB*. For instance, the E334K alteration was detected in LUAD and HNSC. The main mutation of *ACTN4* in GBMLGG is Amplification (0.73%), LGG is Deep Deletion (1.16%) and Amplification (0.78%), PAAD is Amplification (7.57%, 5.98%), LUAD is Amplification (3.49%, 1.41%, 2.61%), MESO is Amplification (2.3%, 1.15%) and Mutation (1.15%), and KIRC is Mutation (0.78%, 0.38%, 0.22%) (Figure 10C). Most substitution mutations were C-to-T transitions (37.09%) (Figure 10D). There were 23 mutations in the full sequence of *ACTN4*. For instance, the D874Rfs*17 alteration was detected in LUAD and KIRC (Supplementary Figure S7B). The main mutation of *INF2* in LIHC is Mutation (1.08%, 0.8%) and Deep Deletion (1.08%), HNSC is Amplification (1.32%, 1.43%, 0.96%), and GBM is Amplification (0.71%, 0.49%) (Figure 10E). Most substitution mutations were C-to-T transitions (41.33%) (Figure 10F). There were 14 mutations in the full sequence of *INF2*. For instance, the K697M alteration was detected in HNSC (Supplementary Figure S7C). The main mutation of *MYL6* in GBMLGG is Deep Deletion and Amplification (0.45%), LGG is Deep Deletion (0.97%, 0.58%), ACC is Amplification (3.3%, 3.26%), and SARC is Amplification (1.53%, 1.18%) (Figure 10G). Most substitution mutations were G-to-A transitions (26.09%) (Figure 10H). There were two mutations in the full sequence of *MYL6*. For instance, the H111R alteration was detected in SARC (Supplementary Figure S7D). The alteration frequency, alteration type, and alteration count of pan-cancer are shown in Supplementary Table S3.

### 3.10. Tumor Pathological Staining

**Figure 11:**
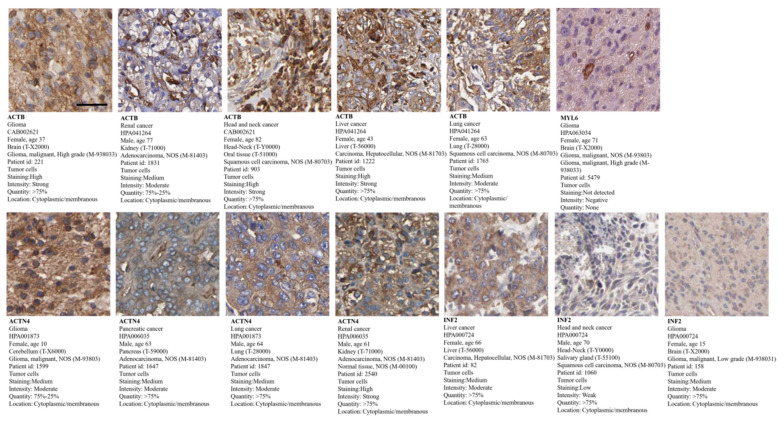
Tumor pathological staining of ACTB in glioma, renal cancer, head and neck cancer, liver cancer, and lung cancer; ACTN4 in glioma, pancreatic cancer, lung cancer, and renal cancer; INF2 in liver cancer, head and neck cancer, and glioma; MYL6 in glioma (scale bar, 20 µm).

We analyzed the pathological staining of proteins in tumor patients using the HPA database and observed that ACTB exhibits strong positivity (>75%) in glioma, head and neck cancer, liver cancer, and lung cancer and moderate positivity (75–25%) in renal cancer. ACTN4 shows strong positivity (>75%) in pancreatic cancer, lung cancer, and renal cancer and moderate positivity (75–25%) in glioma. INF2 shows strong positivity (>75%) in liver cancer, head and neck cancer, and glioma. The expression locations for all mentioned proteins are in the cytoplasmic and membranous regions. However, MYL6 was not detected in glioma (Figure 11). The expression of proteins in normal tissues is shown in Supplementary Figure S8.

### 3.11. Construction of the Gene–miRNA Network

**Figure 12:**
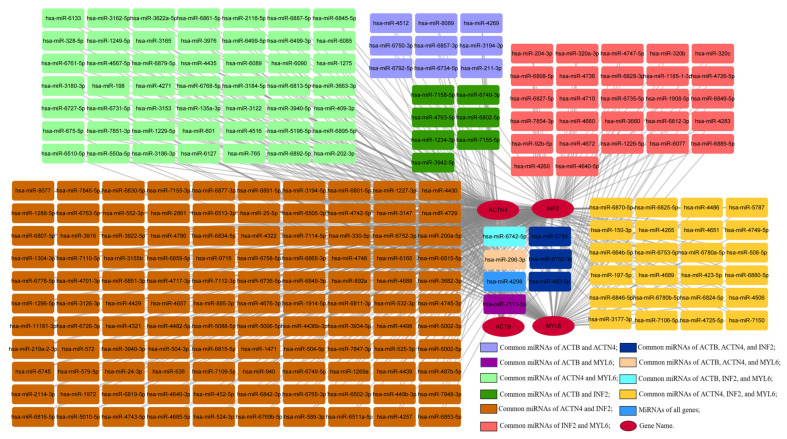
Coexpression network of DEDRGs and target miRNAs.

MicroRNAs (miRNAs) are involved in the occurrence and development of various neurodegenerative diseases such as AD, PD, and Huntington’s disease. In tumors, miRNAs are widely studied and considered important tumor suppressors or promoters. In this analysis, 237 nodes and 497 edges were obtained, among which miRNAs with high degree values include hsa-miR-4298, hsa-miR-296-3p, hsa-miR-150-3p, hsa-miR-493-5p, and hsa-miR-6742-5p (Figure 12). Studies have shown that serum levels of miR-4298 are significantly reduced in GBM patients [28]. Aberrant expression of miR-296 plays an important role in various non-human malignant tumor cellular processes, including cell proliferation, apoptosis, invasion, and epithelial–mesenchymal transition (EMT) [29]. miR-150 acts as a double-edged sword in malignant cells, leading to either tumor suppression or oncogenic functions. Aberrant levels of miR-150 can be detected in metastatic cells closely associated with cancer cell migration, invasion, and angiogenesis [30]. miR-493, as a tumor suppressor miRNA, suppresses cell motility by downregulating RhoC and FZD4 in BLCA. Increased expression of miR-493 prevents liver metastasis of COAD cells and inhibits proliferation and invasion of LIHC. In LUAD, miR-493 inhibits tumor growth, invasion, and metastasis, and forced expression of miR-493 promotes sensitivity to cisplatin chemotherapy. Additionally, miR-493 promotes proliferation, invasion, and chemoresistance in STAD [31]. miR-6742-5p might be a regulator of LUAD progression by targeting the FGF8/ERK1/2/MMPs signaling pathway [32]. miRNA plays an important regulatory role in neurodegenerative diseases and tumors. Studying its changes and functions can help reveal the mechanisms of disease occurrence and provide new ideas and possibilities for the diagnosis and treatment of diseases.

### 3.12. Therapeutic Drugs and Molecular Docking

**Figure 13:**
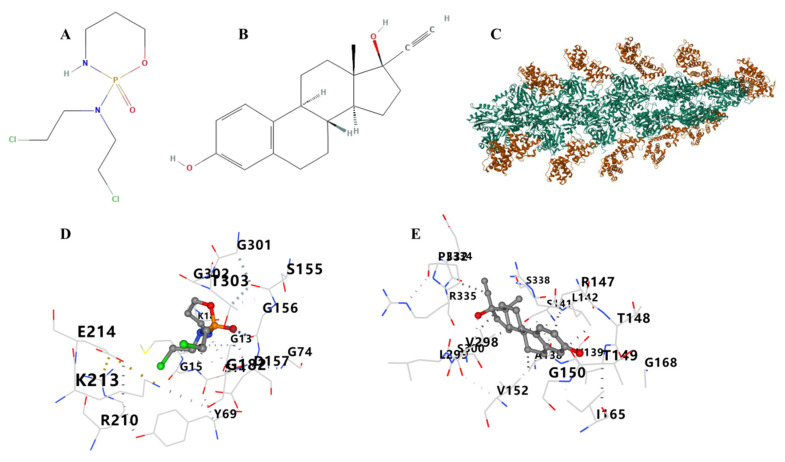
Binding mode of screened drugs to their targets by molecular docking. (**A**) The structure of cyclophosphamide. (**B**) The structure of ethinyl estradiol. (**C**) The structure of ACTB (3byh). (**D**) Molecular docking results of ACTB and cyclophosphamide. (**E**) Molecular docking results of ACTB and ethinyl estradiol.

Therapeutic drugs that target ACTB include cyclophosphamide and ethinyl estradiol. The results showed that a drug candidate is bound to ACTB through visible hydrogen bonds and strong electrostatic interactions (Figure 13). ACTB with cyclophosphamide and ethinyl estradiol had binding energy of −5.5 kcal/mol and −10 kcal/mol, indicating stable binding. Other docking positions of drugs and proteins and energies are shown in Supplementary Figure S9 and Table 2. As shown in Supplementary Figure S10, the ACTN4, INF2, and MYL6 binding pockets were predicted (drug score > 0.5). Supplementary Table S4 shows the volume, surface, drug score, and simple score of binding pockets with higher drug scores.

## 4. Discussion

Disulfidptosis, a recently identified novel form of cell death, is primarily triggered by the accumulation of disulfide bonds, resulting in cytoskeletal collapse and subsequent cell death, which is closely associated with disease progression [33]. The high expression of SLC7A11 (which causes intracellular accumulation of disulfides in SLC7A11) in cells subjected to glucose starvation and lacking a repair mechanism induces disulfide stress, leading to disulfide formation. This represents an atypical form of cell death with a specific underlying mechanism [15]. The activation of disulfide formation may depend on three key factors: (1) high expression of SLC7A11, which imports extracellular cysteine and exports intracellular glutamate, resulting in an increased uptake of extracellular cysteine and excessive accumulation of cysteine within the cell, thereby contributing to disulfide stress in cellular metabolism [17,34]; (2) glucose starvation, which hinders glucose metabolism and produces the reduced form of nicotinamide adenine dinucleotide phosphate (NADPH) through the pentose phosphate pathway (PPP) [35]; (3) abnormal disulfide bonds forming between actin cytoskeletal proteins. When all these conditions are satisfied, there is an excessive accumulation of disulfides, leading to the formation of disulfide bonds between actin cytoskeletal proteins, actin contraction, and detachment from the plasma membrane, ultimately resulting in cell contraction and death [36].

The role of DEDRGs in PD and cancer is also worth paying attention to. DEDRGs are involved in important biological processes such as cell death mechanisms and mitochondrial functions and may play a significant role in the pathogenesis of Parkinson’s disease and cancer. In this study, we found that the biological functions of ACTB, ACTN4, INF2, and MYL6 in PD include the following: NIK/NF-kappaB signaling negative regulation of substrate adhesion-dependent cell spreading, bicellular tight junction assembly, positive regulation of cellular component movement, regulation of mitochondrial fission, regulation of apoptotic process, the peroxisome proliferator-activated receptor signaling pathway, the tumor necrosis factor-mediated signaling pathway, the ephrin receptor signaling pathway, positive regulation of gene expression, epigenetic, and ATP-dependent chromatin remodeling. Subsequently, in the MPTP-induced PD mouse model, we observed a decrease in ACTB expression and an increase in the expression of ACTN4, INF2, and MYL6. Furthermore, by analyzing the GEO datasets, we found that DEDRGs had a diagnostic value (AUC > 0.5), indicating the significant role of DEDRGs in the occurrence and development of PD.

Next, we examined the function of these genes in cancer. β-actin (ACTB) is a highly conserved cytoskeletal protein that is essential for cell growth and migration [37]. ACTB is involved in various cellular processes, such as cell movement, intracellular transport, and signal transduction. Its expression and function are critical for normal cellular dynamics and functionality. ACTB is differentially expressed and plays a significant role in several human diseases, particularly cancer [38,39]. It is well established that the cytoskeletal structure and actin microfilament system can regulate tumor cell adhesion and motility, factors that are vital for tumor growth and metastasis [40]. ACTB has multiple distinct roles, highlighting the versatility of actin and its functions in cell biology. One notable function of ACTB in mitochondrial biology is its involvement in mitochondrial fission, where actin polymerization from the endoplasmic reticulum via the formin INF2 has been demonstrated to stimulate two distinct phases [41]. The silencing of INF2 makes neurons more vulnerable to cell death, while the overexpression of INF2 has a protective effect. Additionally, the F-actin polymerization factor activates INF2. Therefore, dendritic actin recombination induced by ischemia is an intrinsic survival-promoting response that helps protect neurons from cell death caused by edema [26]. The actin-binding protein ACTN4, part of the actin-binding protein family and a non-muscle α-actin, has long been linked to cancer development. Numerous clinical studies have shown that changes in *ACTN4* gene expression are associated with the invasiveness and metastatic potential of certain tumors [40]. Furthermore, ACTN4 is a novel participant in the DNA damage response pathway [42]. ACTN4 has been found to be involved in various cellular processes, including cell migration, cell cycle regulation and growth, EMT, retroviral replication, and activation of transcription factors and nuclear receptors [43]. INF2 is a novel mitochondrial dynamic regulator that is associated with activating mitochondrial fission and myocardial ischemia–reperfusion injury [44,45]. Excessive mitochondrial fission can lead to uneven distribution of mitochondrial DNA in daughter mitochondria [46]. Functionally, the consequences of INF2 activation include the collapse of mitochondrial membrane potential, mitochondrial oxidative stress, and caspase-9 activation [47]. Additionally, INF2 activation can also affect endoplasmic reticulum (ER) stress and mitochondrial calcium homeostasis [48]. MYL6 is the essential light chain within the NM2 complex, thereby involved in regulating the actin cytoskeleton. While the function of ECLs is not clear, they likely play a role in stabilizing the NM2 complex. NM2 contributes to cell motility, and abnormal expression has been reported in various diseases including cancer. In humans, there are three different forms of NM2 (NM2A, NM2B, and NM2C), with MYL6 found to interact with MYH14 and RLC9, 12A, or 12B to form NM2C [49,50]. Low expression of MYL6 can diminish the stability of RLC and MYH chains, resulting in the degradation of NM2 and subsequent deformation of actin filaments, which in turn promotes increased cell migration. Alternatively, it is also possible that in the absence of MYL6, the regulatory effect on the MYH chain is lost, leading to the formation of constitutively active NM2. This could further activate NM2 on actin filaments, thereby enhancing the migration of melanoma cells. It would be intriguing to investigate whether altered MYL6 levels directly affect the protein levels of other ELCs or RLCs and whether MYL6 knockdown significantly modifies the architecture of the actin cytoskeleton [50].

Our study showed that these genes were more highly infiltrated in Macrophages, NK cells, Neutrophils, Eosinophils, CD8 T cells, T cells, T helper cells, B cells, dendritic cells, and mast cells in pan-cancer patients. Most substitution mutations were G-to-A transitions and C-to-T transitions. We also found that miR-4298, miR-296-3p, miR-150-3p, miR-493-5p, and miR-6742-5p play important roles in cancer and PD. miRNAs can impact the function and survival of neuronal cells by regulating the expression of target genes [51,52]. For example, some miRNAs are overexpressed or underexpressed in neurodegenerative diseases, leading to abnormal expression or functional disruption of relevant genes, thereby triggering pathological changes [53,54]. miRNAs can influence tumor growth, invasion, metastasis, and treatment response by regulating oncogenes [55,56,57]. Further research on the functions and regulatory mechanisms of miRNAs helps deepen the understanding of the pathological processes of these diseases and provides new perspectives and methods for the diagnosis, treatment, and prevention of related diseases.

Artificial intelligence (AI) can tailor treatment plans for individual patients, optimizing efficacy and minimizing side effects. It continuously monitors treatment response and adjusts therapies, promoting precision medicine. However, access to high-quality molecular data and advanced diagnostic tools may be limited in some settings. Personalized medicine can be expensive, but AI can simulate experiments to predict outcomes and optimize protocols, saving time and resources. It can also identify the most impactful experiments, reducing the need for animal testing. In silico models are not perfect and require validation in real-world experiments. AI-powered simulations can analyze cellular processes related to disulfidptosis with high resolution, aiding in hypothesis generation. The accuracy of these simulations depends on the quality of the biological models and requires computational resources and expertise. AI should complement traditional methods, not replace them. A collaborative approach involving researchers, clinicians, and data scientists is essential to ensure AI applications are biologically grounded, ethical, and practical [58,59,60,61,62].

Research on “disulfidptosis” (disulfide-induced cell death), while potentially limited in its direct application to specific neurodegenerative diseases or cancer types, offers significant insights and promising avenues for the study of other disease categories. In AD, notable oxidative stress and mitochondrial dysfunction are prevalent, and disulfides, through their antioxidant or mitochondrial regulatory functions, may impact neuronal death in AD [63]. One of the hallmarks of AD is the aggregation of β-amyloid and tau proteins, which may contribute to cell death [64]. Investigating whether disulfides can influence the aggregation or clearance of these proteins could lead to novel therapeutic approaches for AD. PD is characterized by the progressive death of dopaminergic neurons in the substantia nigra. The mechanism of disulfide-induced cell death may share similarities with neuronal death in PD, rendering its study in PD models potentially valuable. Additionally, the aggregation of α-synuclein is another crucial pathological process in PD. Exploring whether disulfides affect the aggregation, propagation, or clearance of α-synuclein could provide new clues for PD treatment strategies [65]. Huntington’s disease (HD) arises from the expansion of CAG repeats in the Huntingtin gene, leading to the aggregation of mutant Huntingtin protein and neuronal death [66]. Research into whether disulfides impact the toxicity of mutant Huntingtin protein could reveal novel therapeutic targets.

Many types of cancer cells exhibit metabolic reprogramming, such as enhanced glycolysis and glutamine metabolism [67]. Disulfides may influence cancer cell growth and survival by modulating these metabolic pathways. Based on the mechanism of disulfide-induced cell death, developing anticancer drugs targeting specific metabolic pathways could emerge as a new therapeutic strategy. Some cancer cells are resistant to apoptotic signals, enabling them to evade immune clearance and proliferate continuously. Investigating whether disulfides induce cancer cell death through non-apoptotic pathways, such as disulfide-induced cell death, could offer new methods for overcoming apoptosis resistance. Cancer-associated fibroblasts (CAFs) play a crucial role in the tumor microenvironment, supporting cancer cell growth and invasion [68]. Studying whether disulfides affect CAF function or their interactions with cancer cells could identify new therapeutic targets for cancer treatment. While research on the mechanism of disulfide-induced cell death in specific disease models is still preliminary, its potential antioxidant, mitochondrial regulatory, protein-aggregation-modulating, and metabolic reprogramming properties offer broad prospects for the study of other neurodegenerative diseases and cancer types. Future research should further explore the mechanisms of disulfides in these diseases and assess their potential as therapeutic strategies.

A notable potential application of the disulfidptosis mechanism lies in disease treatment. By inducing oxidative stress and cell death in tumor cells, disulfidptosis can serve a therapeutic function in cancer therapy. The enhanced processes of lamellipodia and invasive pseudopodia may make metastatic cancer cells more susceptible to disulfidptosis [69]. Beyond cardiovascular and neurological disorders, sulfur-induced cell death is also regarded as a significant therapeutic target related to lamellipodia. In conditions like myocardial infarction and stroke, inhibiting disulfidptosis has been demonstrated to mitigate damage. Therefore, disulfidptosis represents a very promising mechanism for disease treatment, warranting further research and exploration. The identification of *ACTB*, *ACTN4*, *INF2*, and *MYL6* as DEDRGs that are implicated in both PD and various cancer types opens up exciting possibilities for developing novel therapeutic strategies for human patients. Here, we expand on the potential therapeutic implications of our findings: Given the role of these DEDRGs in regulating cellular processes such as apoptosis, mitochondrial function, and the NF-κB signaling pathway, they represent potential therapeutic targets. For example, modulating the expression or activity of these genes through small molecules, gene therapy, or RNA interference could potentially slow disease progression or enhance the effectiveness of existing treatments. The differential expression patterns of these genes across different cancer types and subtypes suggest that precision medicine approaches could be developed. By profiling the expression of these DEDRGs in individual patients, it may be possible to tailor treatment regimens that are specifically targeted to the molecular characteristics of their disease. Our findings that these genes are associated with immune cell infiltration in cancer patients suggest that immunotherapeutic strategies may be particularly effective. Boosting the immune system’s ability to target cancer cells that are expressing these DEDRGs could enhance treatment outcomes. The identification of miRNAs such as miR-4298, miR-296-3p, miR-150-3p, miR-493-5p, and miR-6742-5p as potential regulators of these DEDRGs in both PD and cancer suggests that existing drugs that target these miRNAs or their downstream pathways could be repurposed for treating these diseases. The expression levels of these DEDRGs could serve as biomarkers for the early detection of PD and cancer, as well as for predicting disease progression and response to treatment. By monitoring these biomarkers in patients, clinicians could make more informed decisions about treatment options and adjust them as needed. Given the complexity of both PD and cancer, it is likely that targeting multiple pathways simultaneously will be necessary to achieve optimal therapeutic outcomes. Our findings suggest that combining therapies that target these DEDRGs with other drugs or treatments that address different aspects of the disease may yield better results. Finally, our results provide valuable insights into the molecular mechanisms underlying the relationship between PD and cancer, which could inform the development of novel drugs specifically designed to target these DEDRGs. Such drugs could potentially have broad applications in the treatment of neurodegenerative diseases and cancers. In conclusion, our study highlights the potential of *ACTB*, *ACTN4*, *INF2*, and *MYL6* as therapeutic targets and biomarkers for PD and cancer. Further research into the roles of these genes and their downstream pathways is needed to fully realize their therapeutic potential and develop effective treatments for these devastating diseases.

## Figures and Tables

**Table 1:** The full name and abbreviation of cancer.

Cancer	Abbreviation
Adrenocortical Cancer	ACC
Bladder Cancer	BLCA
Breast Cancer	BRCA
Cervical Cancer	CESC
Bile Duct Cancer	CHOL
Colon Cancer	COAD
Colon and Rectal Cancer	COADREAD
Large B-cell Lymphoma	DLBC
Esophageal Cancer	ESCA
FFPE Pilot Phase II	FPPP
Glioblastoma	GBM
Lower Grade Glioma and Glioblastoma	GBMLGG
Head and Neck Cancer	HNSC
Kidney Chromophobe	KICH
Kidney Clear Cell Carcinoma	KIRC
Kidney Papillary Cell Carcinoma	KIRP
Acute Myeloid Leukemia	LAML
Lower Grade Glioma	LGG
Liver Cancer	LIHC
Lung Adenocarcinoma	LUAD
Lung Cancer	LUNG
Lung Squamous Cell Carcinoma	LUSC
Mesothelioma	MESO
Ovarian Cancer	OV
Pancreatic Cancer	PAAD
Pan-Cancer	PANCAN
Pheochromocytoma and Paraganglioma	PCPG
Prostate Cancer	PRAD
Rectal Cancer	READ
Sarcoma	SARC
Melanoma	SKCM
Stomach Cancer	STAD
Testicular Cancer	TGCT
Thyroid Cancer	THCA
Thymoma	THYM
Endometrioid Cancer	UCEC
Uterine Carcinosarcoma	UCS
Ocular melanomas	UVM

**Table 2:** The results of protein–drug molecular docking, including Vina score and contact residues.

	CurPocket ID	Vina Score	Contact Residues
ACTB–cyclophosphamide	C3	−5.5	Chain A: ASP11 GLY13 SER14 GLY15 MET16 LYS18 TYR69 GLY74 GLN137 SER155 GLY156 ASP157 GLY182 ARG183 ARG210 LYS213 GLU214 GLY301 GLY302 THR303 MET305 TYR306 LYS336
	C2	−5.4	Chain A: ASP11 ASN12 GLY13 SER14 GLY15 MET16 LYS18 TYR69 GLY74 GLU107 ALA108 PRO109 LEU110 ASN111 PRO112 LYS113 ARG116 ILE136 GLN137 ALA138 VAL139 SER141 LEU142 SER145 GLY146 ARG147 THR149 GLY150 VAL152 ASP154 SER155 GLY156 ASP157 HIS161 ILE165 PRO172 ILE175 ARG177 GLY182 ARG183 VAL298 LEU299 SER300 GLY301 GLY302 THR303 ILE330 PRO332 ARG335 LYS336 SER338 VAL339 HIS371 CYS374 PHE375
	C1	−5.0	Chain A: ASP3 ASP4 PHE21 ARG28 ALA29 TYR91 ASN92 GLU93 LEU94 ARG95 VAL96 ALA97 PRO98 GLU99 GLU100 Chain B: ASP394 CYS397 TYR398 LEU466 LYS467 PHE468 LYS483 LYS484 LEU487 GLY488 LEU489 TRP491 GLN492 ARG495 PHE496 LEU499 GLN500 LEU502 LYS503 ARG506 MET514 THR515 ASP516 ALA517 VAL604 ASN605 GLN606 LYS607
	C5	−4.0	Chain B: ASP408 SER409 TYR410 ASN412 GLU416 ASP417 VAL418 ARG419 ASN420 TRP422 ILE423 GLU426 PRO444 PRO445 ILE446 LYS447 LYS452 VAL479
	C4	−3.6	Chain B: ASP408 SER409 TYR410 ASN412 GLU416 ASP417 VAL418 ARG419 ASN420 TRP422 ILE423 GLU426 PRO444 PRO445 ILE446 LYS447 LYS452 VAL479
ACTB–ethinyl estradiol	C2	−10.0	Chain A: ASP11 ASN12 GLY13 SER14 GLY15 MET16 LYS18 TYR69 HIS73 GLU107 ALA108 PRO109 LEU110 ASN111 PRO112 LYS113 ARG116 ILE136 GLN137 ALA138 VAL139 SER141 LEU142 SER145 GLY146 ARG147 THR148 THR149 GLY150 ILE151 VAL152 SER155 GLY156 ASP157 HIS161 ILE165 GLY168 PRO172 ILE175 ARG177 GLY182 ARG183 THR186 LYS213 GLU214 VAL298 LEU299 SER300 GLY301 GLY302 THR303 PRO332 PRO333 GLU334 ARG335 LYS336 TYR337 SER338 VAL339 HIS371 ARG372 CYS374 PHE375
	C3	−8.3	Chain A: ASP11 ASN12 GLY13 SER14 GLY15 MET16 LYS18 TYR69 GLN137 SER155 GLY156 ASP157 GLY182 ARG183 LEU185 THR186 ARG210 LYS213 GLU214 LYS215 CYS217 GLY301 GLY302 THR303 MET305 TYR306 PRO307 LYS336 TYR337 SER338 VAL339
	C5	−6.0	Chain B: ASP408 SER409 TYR410 VAL411 ASN412 GLU416 ASP417 VAL418 ARG419 ASN420 TRP422 ILE423 GLU426 LYS443 PRO444 PRO445 ILE446 LYS452 VAL479
	C4	−5.6	Chain A: GLN59 SER60 LYS61 ARG62 GLY63 ILE64 GLY197 TYR198 SER199 PHE200 THR202 THR203 ALA204 GLU205 ILE208 ASP211 LYS215 TYR240 LEU242 PRO243 ASP244 GLN246 VAL247 ILE248

## Data Availability

The datasets presented in this study can be found in the online repositories. The names of the repository/repositories and accession numbers can be found in the article/Supplementary Materials.

## Supplementary Materials

The following supporting information can be downloaded at https://www.mdpi.com/article/10.3390/cimb46090600/s1: Figure S1: Venn diagram of overlapping genes between DEGs and DRGs; Figure S2: Expression of (A) ACTB, (B) ACTN4, (C) INF2, and (D) MYL6 in 31 primary tissues retrieved from GTEx; Figure S3: The proteins are expressed differently in various tissues during fetal and adulthood, including (A) ACTB, (B) ACTN4, (C) INF2, and (D) MYL6; Figure S4: Survival analysis of DEDRGs expression in pan-cancer from GEPIA database; Figure S5: Survival analysis of DEDRGs expression in pan-cancer. (A–G) Survival curves of ACTB in KIPAN, KICH, LAML, GBM, PAAD, ACC, and OV. (H) Survival curves of ACTN4 in LAML. (I,J) Survival curves of INF2 in LUAD and UVM. (K–O) Survival curves of MYL6 in STAD, MESO, KIRC, THYM, and OV; Figure S6: Single cell tissues overview of DEDRGs, including (A) ACTB, (B) ACTN4, (C) INF2, and (D) MYL6; Figure S7: Main mutation types of (A) ACTB, (B) ACTN4, (C) INF2, and (D) MYL6; Figure S8: The expression of proteins in normal tissues, including the expression of ACTB in cerebral cortex, kidney, liver, and lung; the expression of ACTN4 in cerebral cortex, pancreas, lung, and kidney; the expression of INF2 in liver and cerebral cortex; the expression of MYL6 in cerebral cortex (Scale bar, 20 µm). Figure S9: Binding mode of screened drugs to their targets by molecular docking. (A–D) Molecular docking results of ACTB and cyclophosphamide. (E–G) Molecular docking results of ACTB and ethinyl estradiol; Figure S10: Prediction of protein-binding pockets with drug-binding scores. (A) The structure of ACTN4 (2r0o). (B) The structure of INF2 (q27j81). (C) The structure of MYL6 (p60660). (D) Prediction of ACTN4 protein binding pocket. (E) Prediction of INF2 protein binding pocket. (F) Prediction of MYL6 protein binding pocket; Table S1:  The expression values of tumor samples and normal samples; Table S2: Tumor expression data and overall survival data of samples; Table S3: Alteration frequency, alteration type, and alteration count of pan-cancer; Table S4: The volume, surface, drug score, and simple score of binding pockets with higher drug scores.
