# Supplementary material for: Disulfidptosis: A New Target for Parkinson’s Disease and Cancer
Source: Curr Issues Mol Biol. 2024 Sep 12;46(9):10038–64. doi: 10.3390/cimb46090600 (PMC11430384; doi:10.3390/cimb46090600)
Supplement: Supplementary file 1 [file cimb-46-00600-s001.zip › Supplementary materials.pdf]

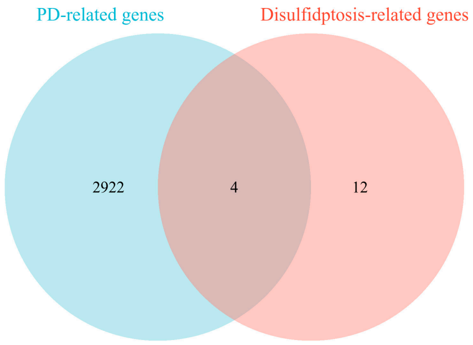

Figure S1 Venn diagram of overlapping genes between DEGs and DRGs.

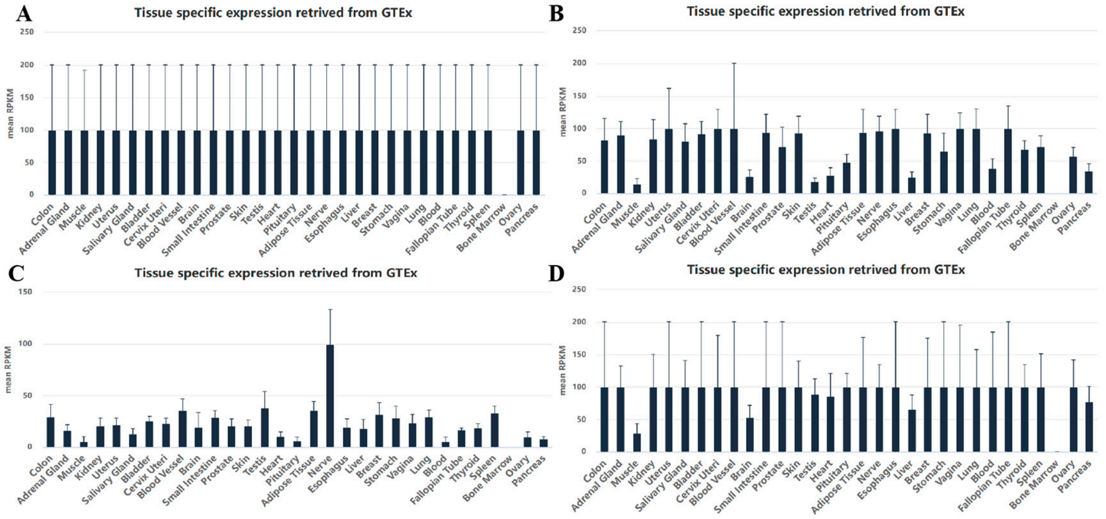

Figure S2 Expression of (A) ACTB, (B) ACTN4, (C) INF2, and (D) MYL6 in 31 primary tissues retrieved from GTEx.

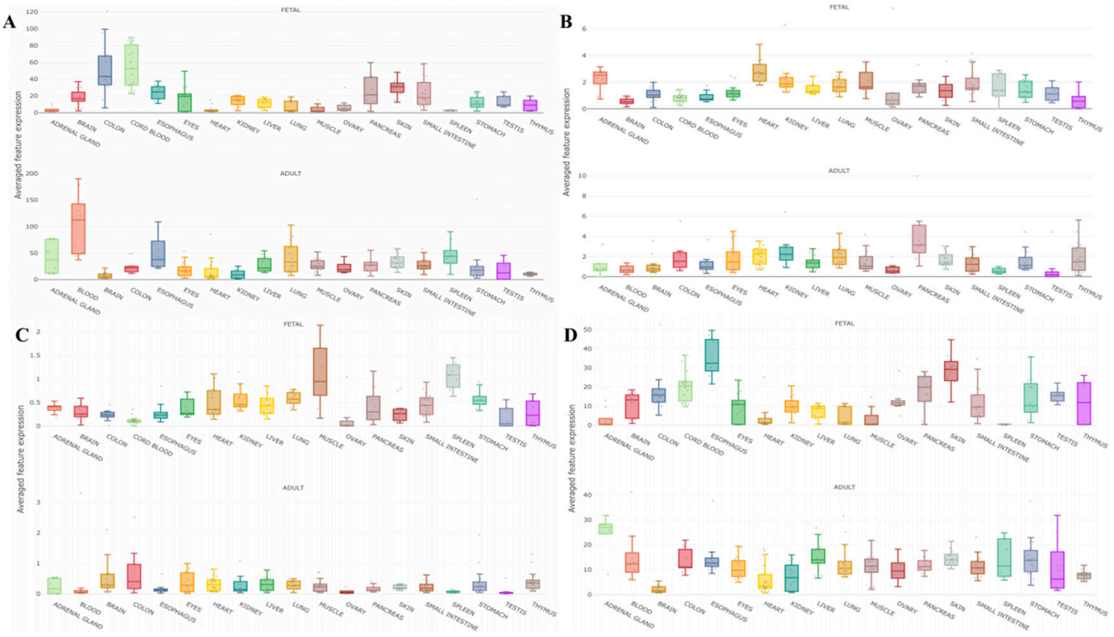

Figure S3 The proteins are expressed differently in various tissues during fetal and adulthood, including (A) ACTB, (B) ACTN4, (C) INF2, and (D) MYL6.

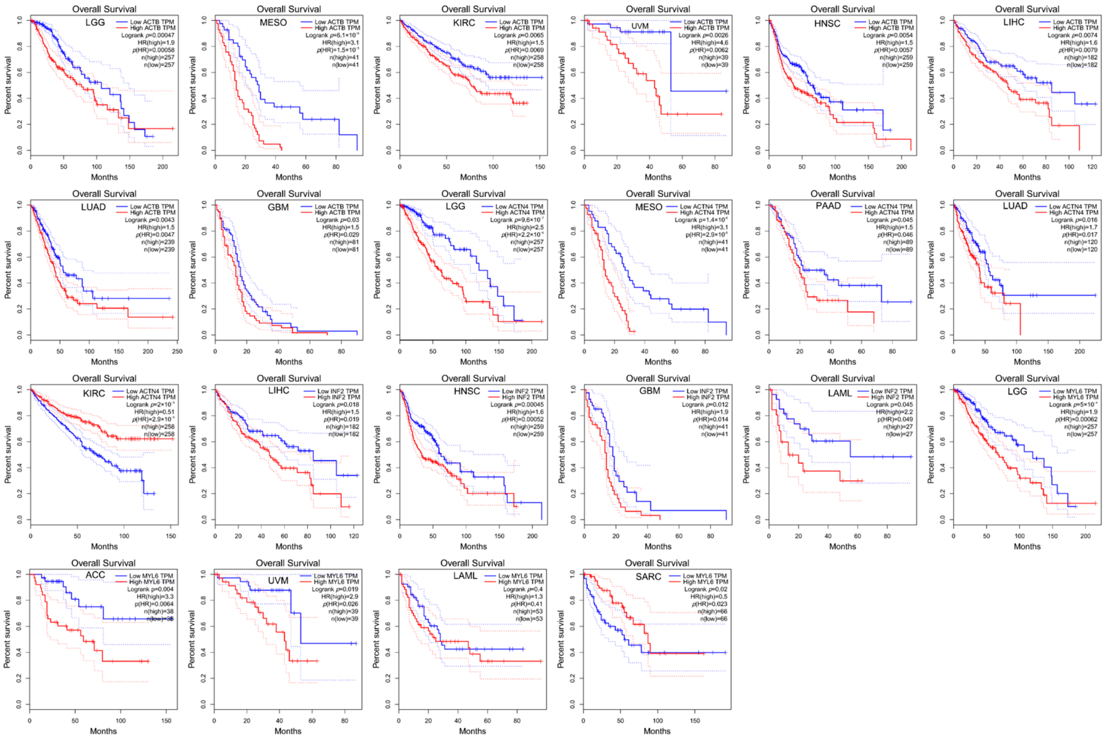

Figure S4 Survival analysis of DEDRGs expression in pan-cancer from GEPIA database.

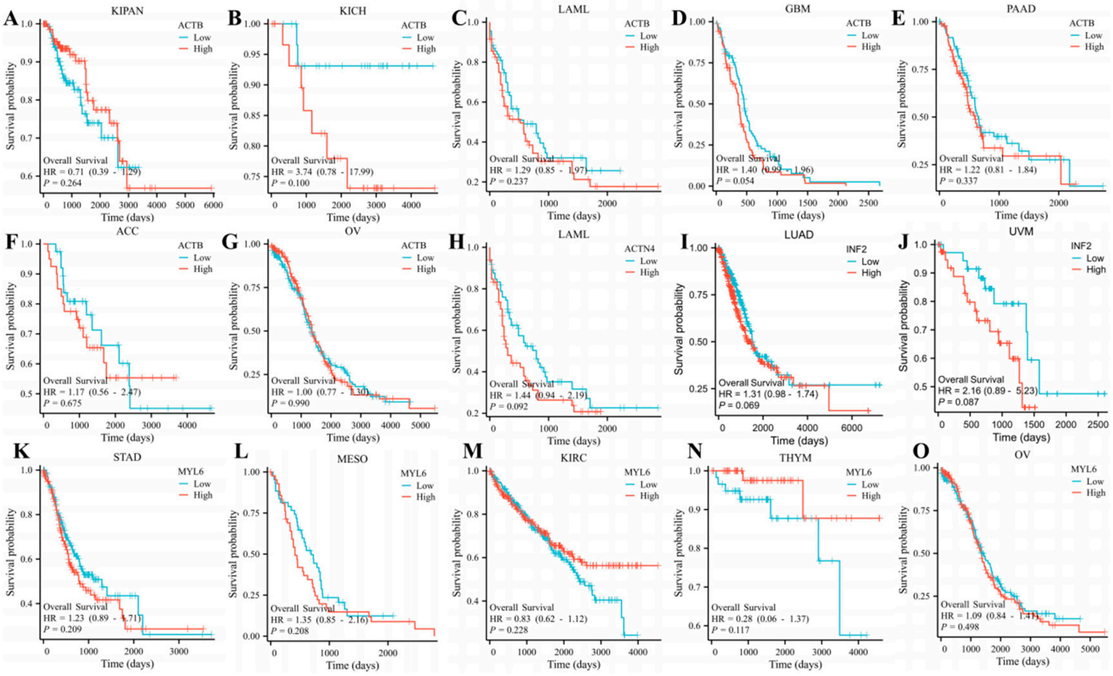

Figure S5 Survival analysis of DEDRGs expression in pan-cancer. (A-G) Survival curves of ACTB in KIPAN, KICH, LAML, GBM, PAAD, ACC, and OV. (H) Survival curves of ACTN4 in LAML. (I-J) Survival curves of INF2 in LUAD and UVM. (K-O) Survival curves of MYL6 in STAD, MESO, KIRC, THYM, and OV.

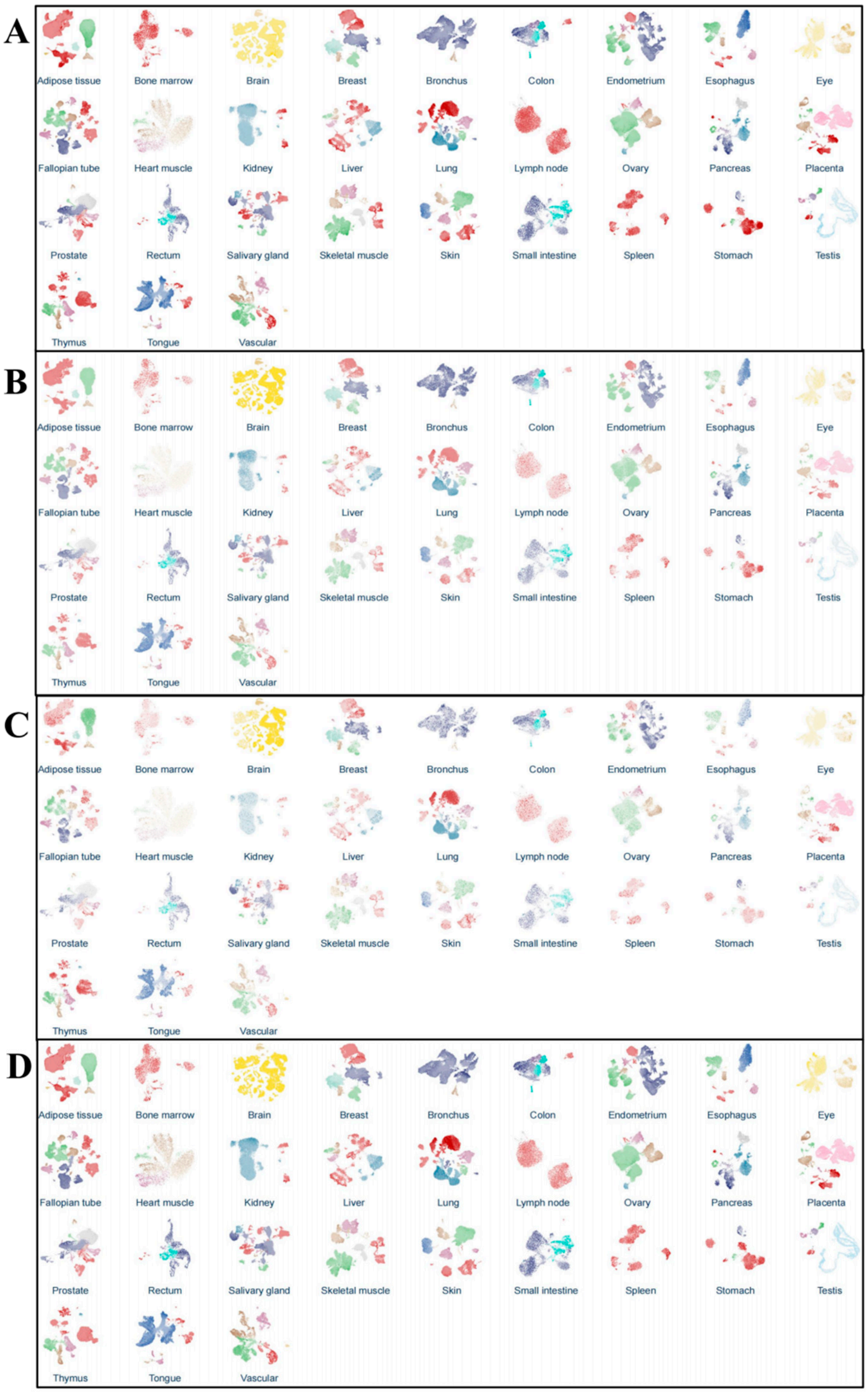

Figure S6 Single cell tissues overview of DEDRGs, including (A) ACTB, (B) ACTN4, (C) INF2, and (D) MYL6.

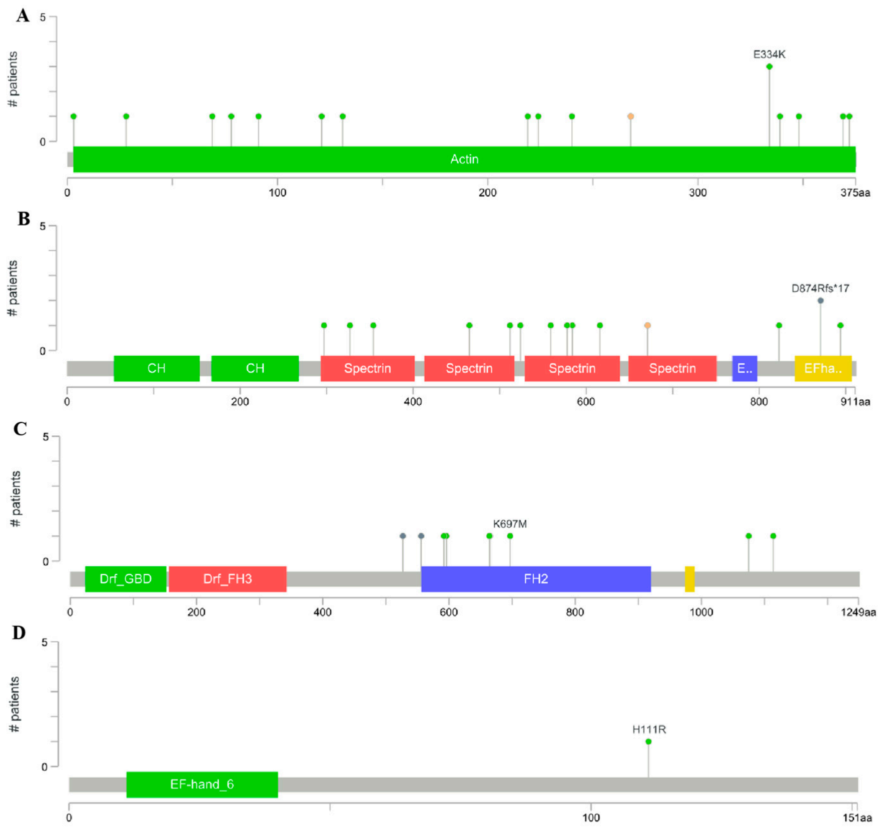

Figure S7 Main mutation types of (A) ACTB, (B) ACTN4, (C) INF2, and (D) MYL6.

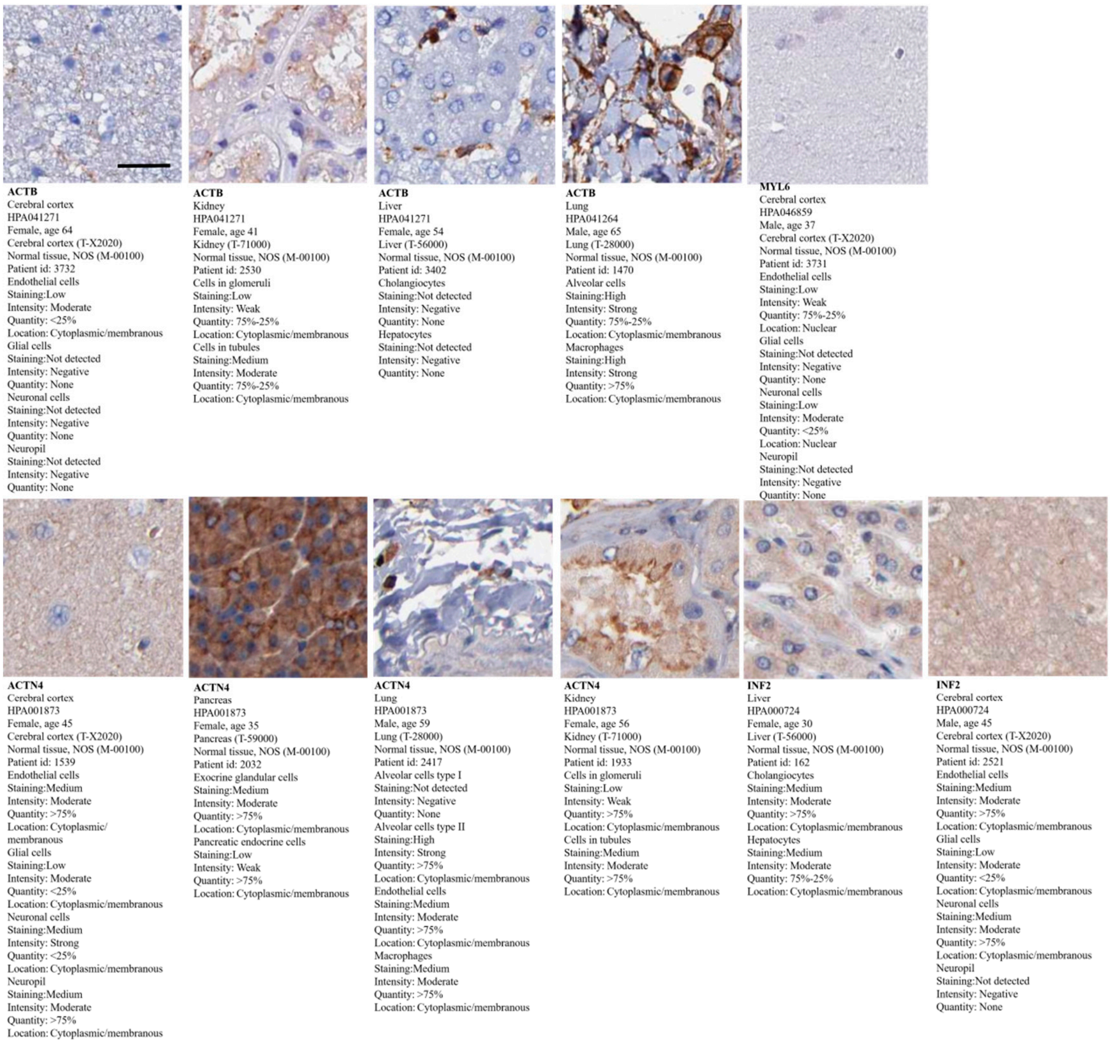

Figure S8 The expression of proteins in normal tissues, including the expression of ACTB in cerebral cortex, kidney, liver, and lung; the expression of ACTN4 in cerebral cortex, pancreas, lung, and kidney; the expression of INF2 in liver and cerebral cortex; the expression of MYL6 in cerebral cortex (Scale bar, 20 μm)..

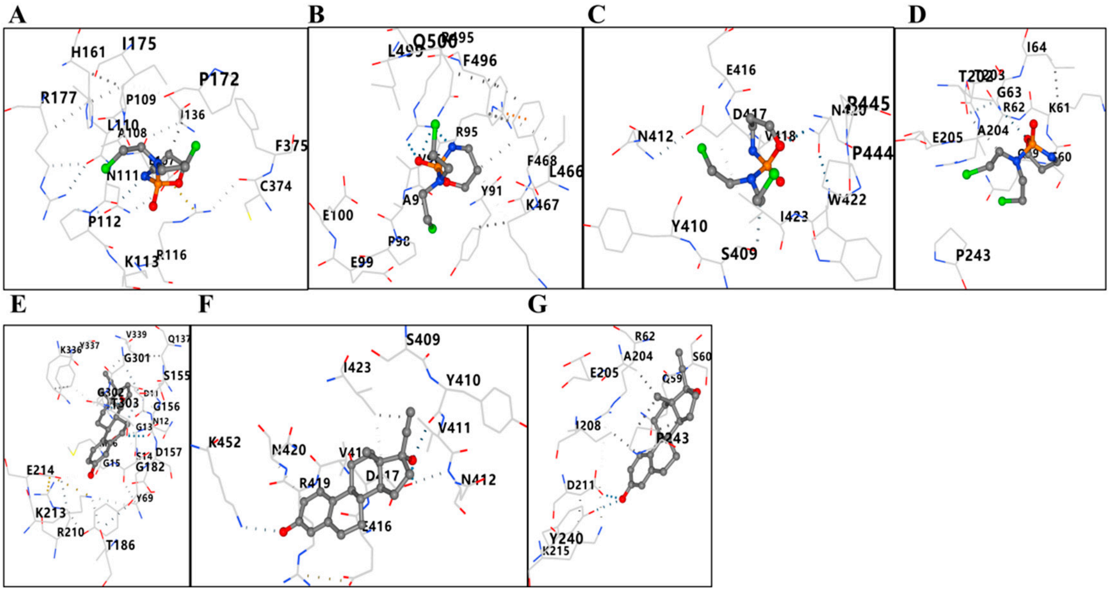

Figure S9 Binding mode of screened drugs to their targets by molecular docking. (A-D) Molecular docking results of ACTB and cyclophosphamide. (E-G) Molecular docking results of ACTB and ethinyl estradiol.

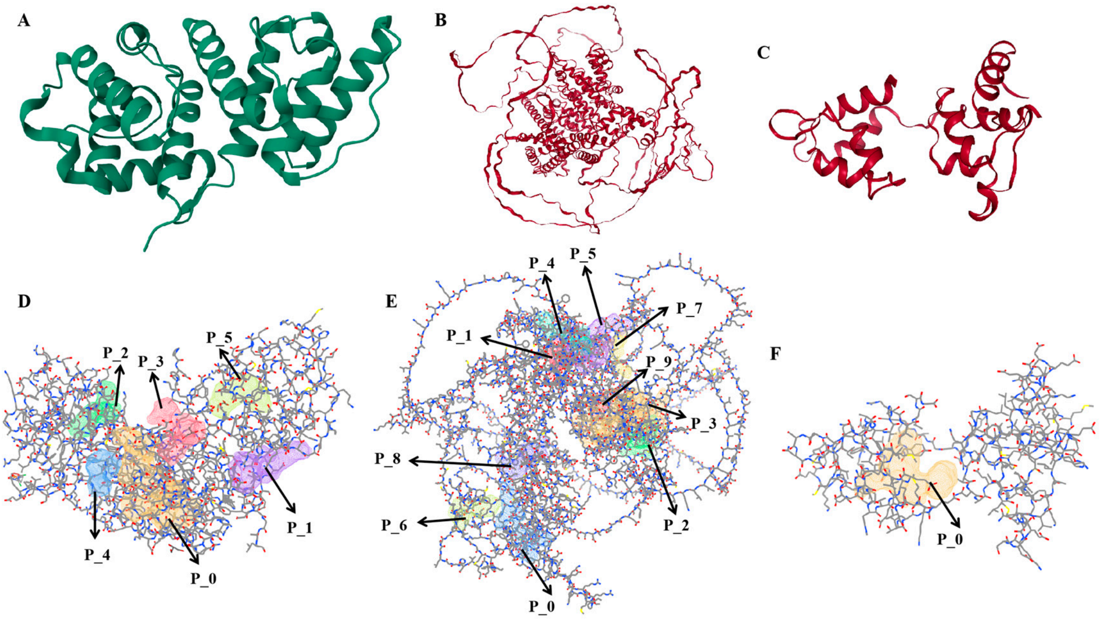

Figure S10 Prediction of protein-binding pockets with drug-binding scores. (A) The structure of ACTN4 (2r0o). (B) The structure of INF2 (q27j81). (C) The structure of MYL6 (p60660). (D) Prediction of ACTN4 protein binding pocket. (E) Prediction of INF2 protein binding pocket. (F) Prediction of MYL6 protein binding pocket.
